# Arabidopsis ICK/KRP cyclin-dependent kinase inhibitors function to ensure the formation of one megaspore mother cell and one functional megaspore per ovule

**DOI:** 10.1371/journal.pgen.1007230

**Published:** 2018-03-07

**Authors:** Ling Cao, Sheng Wang, Prakash Venglat, Lihua Zhao, Yan Cheng, Shengjian Ye, Yuan Qin, Raju Datla, Yongming Zhou, Hong Wang

**Affiliations:** 1 National Key Laboratory of Crop Genetic Improvement, College of Plant Science and Technology, Huazhong Agricultural University, Wuhan, China; 2 Dept. of Biochemistry, University of Saskatchewan, Saskatoon, SK, Canada; 3 National Research Council Canada, Saskatoon, SK, Canada; 4 Fujian Provincial Key Laboratory of Haixia Applied Plant Systems Biology, Center for Genomics and Biotechnology, Fujian Agriculture and Forestry University, Fuzhou, Fujian, China; Peking University, CHINA

## Abstract

In most plants, the female germline starts with the differentiation of one megaspore mother cell (MMC) in each ovule that produces four megaspores through meiosis, one of which survives to become the functional megaspore (FM). The FM further develops into an embryo sac. Little is known regarding the control of MMC formation to one per ovule and the selective survival of the FM. The ICK/KRPs (interactor/inhibitor of cyclin-dependent kinase (CDK)/Kip-related proteins) are plant CDK inhibitors and cell cycle regulators. Here we report that in the ovules of Arabidopsis mutant with all seven *ICK/KRP* genes inactivated, supernumerary MMCs, FMs and embryo sacs were formed and the two embryo sacs could be fertilized to form two embryos with separate endosperm compartments. Twin seedlings were observed in about 2% seeds. Further, in the mutant ovules the number and position of surviving megaspores from one MMC were variable, indicating that the positional signal for determining the survival of megaspore was affected. Strikingly, ICK4 fusion protein with yellow fluorescence protein was strongly present in the degenerative megaspores but absent in the FM, suggesting an important role of ICKs in the degeneration of non-functional megaspores. The absence of or much weaker phenotypes in lower orders of mutants and complementation of the septuple mutant by *ICK4* or *ICK7* indicate that multiple ICK/KRPs function redundantly in restricting the formation of more than one MMC and in the selective survival of FM, which are critical to ensure the development of one embryo sac and one embryo per ovule.

## Author summary

In most plants, the female germline starts with the differentiation of one megaspore mother cell (MMC) in each ovule that produces multiple megaspores through meiosis. One of the megaspores in a fixed position survives to become the functional megaspore (FM) while the other megaspores undergo degeneration. The FM further develops into an embryo sac. We have been working on the functions and regulation of a family of plant cyclin-dependent kinase inhibitors called ICKs or KRPs. We observed that in the ovules of Arabidopsis mutant with all seven *ICK/KRP* genes inactivated, multiple MMCs, FMs and embryo sacs were formed, and the embryo sacs could be fertilized to produce two embryos with separate endosperm compartments. Further, in mutant ovules the number and position of surviving megaspores from one MMC were variable and ICK4-YFP (yellow fluorescence protein) fusion protein was strongly expressed in the degenerative megaspores but absent in the FM. Those findings together with other results in our study indicate that multiple ICK/KRPs function redundantly in controlling the formation of one MMC per ovule and also in the degeneration of non-functional megaspores, which are critical for the subsequent development of one embryo sac per ovule and one embryo per seed.

## Introduction

The cyclin-dependent kinase (CDK) inhibitors are proteins of usually small molecular masses able to inhibit CDKs through direct binding. Since CDKs are central to cell cycle regulation in eukaryotes, CDK inhibitors are important cell cycle regulators. The ICK/KRP family of plant CDK inhibitors was initially discovered in Arabidopsis and share limited similarity in the C-terminal region with the mammalian Kip/Cip family of CDK inhibitors [1,2,3]. There are seven *ICK/KRP* genes in Arabidopsis [2]. Apart from the C-terminal conserved regions, plant ICK/KRP inhibitors differ at the protein sequence level greatly from the animal Kip/Cip CDK inhibitors and also among themselves, implying possible functional differences. *ICK*/*KRP* genes are present in the genomes of all seed plants examined but absent from bryophytes and algae, and sequence analysis suggests that the plant ICK/KRP family and animal KIP/CIP family might have evolved independently [4].

The CDK inhibitory function of the ICK/KRP CDK inhibitors has been demonstrated both *in vitro* [1,3,5] and *in vivo*, mostly with Arabidopsis ICK/KRPs [2,6,7]. Although the specificity of ICK/KRP interactions with different CDKs and cyclins has not been fully understood, available experimental evidence suggests that ICK/KRP proteins target mostly CDK complexes consisted of the A-type CDK and D-type cyclins [8,9]. There are two conserved motifs in ICK/KRPs responsible for the interactions with the CDK and cyclins [4,10,11]. It has been shown that ICK/KRPs are unstable proteins [6,12,13], and the N-terminal sequence of ICK1 play a key role in regulating the instability as its removal resulted in a dramatic increase in the abundance of GFP-ICK1 fusion protein [6]. Previous results have implicated several components of the ubiquitin proteasome system (UPS) for their involvement in the degradation of ICK/KRP proteins and in particular a role by the SCF-mediated protein ubiquitination [14,15,16,17,18]. However, the specific mechanistic details for the ubiquitin-mediated degradation of ICK/KRPs and the signal sequences in ICK/KRP proteins remain unknown.

A number of studies have shown that constitutive overexpression of an *ICK*/*KRP* gene can have dramatic effects on plant growth and morphology, including reduced cell numbers, smaller plant sizes and serrated leaves in Arabidopsis [2,7,19,20]. In rice, plants overexpressing an *ICK*/*KRP* gene also display a smaller size, reduced seed set and other cellular changes accompanied by increased cell sizes [21,22]. Since an ICK/KRP inhibitor modulates CDK enzymatic activity through direct protein binding, the concentration or level of the ICK/KRP protein is likely important for its function. Indeed, it has been observed that the severity of the phenotypes depends on the expression level of the transgenic ICK/KRP [6]. In addition, tissue-specific expression of Arabidopsis *ICK1* has been shown to restrict cell proliferation of a particular organ [23] or cell type [24].

Knockout or down-regulation could provide more insightful information on the functions of different ICK/KRPs. However, partly due to a possible overlap in functions among ICK/KRPs only a few studies were reported. Knockout of *ICK2/KRP2* was found to promote formation of lateral roots in Arabidopsis [25]. A more recent study investigated a series *ICK/KRP* mutants from single to a quintuple mutant and only observed relatively mild changes in the quadruple and quintuple mutants, including increased seedling growth, sizes of the first two pairs of leaves and seed size as well as increased cell numbers [26]. Those changes became more prominent with increased number of *ICK/KRP* genes inactivated, suggesting that ICK/KRPs function redundantly in plants. Interestingly, inactivation of *ICK3/KRP5* resulted in reduced endoreduplication and cell expansion in roots [27,28].

The phenotypic changes from *ICK/KRP* down-regulation observed in the previous studies were relatively mild and often appear beneficial in terms of plant growth, raising a question regarding why seven *ICK/KRP* genes are needed in plants. The fact that all *ICK/KRP* genes have been retained during evolution and expressed in Arabidopsis suggests that each of the seven *ICK/KRP* genes is required in normal plants and there is a positive selection pressure for maintaining them. We hypothesize that additional important functions for the *ICK/KRP* genes remain to be discovered. To that end, we created the septuple mutant in which all seven *ICK/KRP* genes were inactivated and in this unique background uncovered surprising functions of ICK/KRPs in controlling the number of megaspore mother cell and also the survival of megaspores during the female gametophyte development.

## Results

### Phenotyping of Arabidopsis septuple mutant with seven *ICK*/*KRP* genes down-regulated

We reported phenotypic changes from down-regulating five *ICK*/*KRP* genes in the *ick1 ick2*, *ick5 ick6 ick7* mutant [26]. The overall phenotypes are relatively mild, raising a question regarding why multiple *ICK*/*KRP* genes are needed in seed plants. To unlock possible additional functions, higher orders of *ick* mutants were needed. Thus, we obtained *ick3*/*krp5* (Salk_053537/053533) and *ick4*/*krp6* (Sail_548_B03) mutants (referred to as *ick3-1* and *ick4-1*), and confirmed that the expression of each gene was disrupted (**S1 Fig**). Crosses were made with the *ick* quintuple mutant to create a hextuple (containing six *ICK* mutant alleles except *ICK4*) and septuple mutant (*ick1 ick2 ick3 ick4 ick5 ick6 ick7*) (S2 Fig). All seven *ICK/KRP* genes were inactivated in the septuple mutant (S3 Fig). For convenience, the double and multiple mutants are referred to hereafter simply by the *ick* mutant numbers. For instance, the hextuple i*ck1 ick2 ick3 ick5 cik6 ick7* mutant is referred to as *ick123567* mutant. The specific T-DNA lines used for *ICK1*, *ICK2*, *ICK5*, *ICK6* and *ICK7* were described in [26].

**Fig 1:**
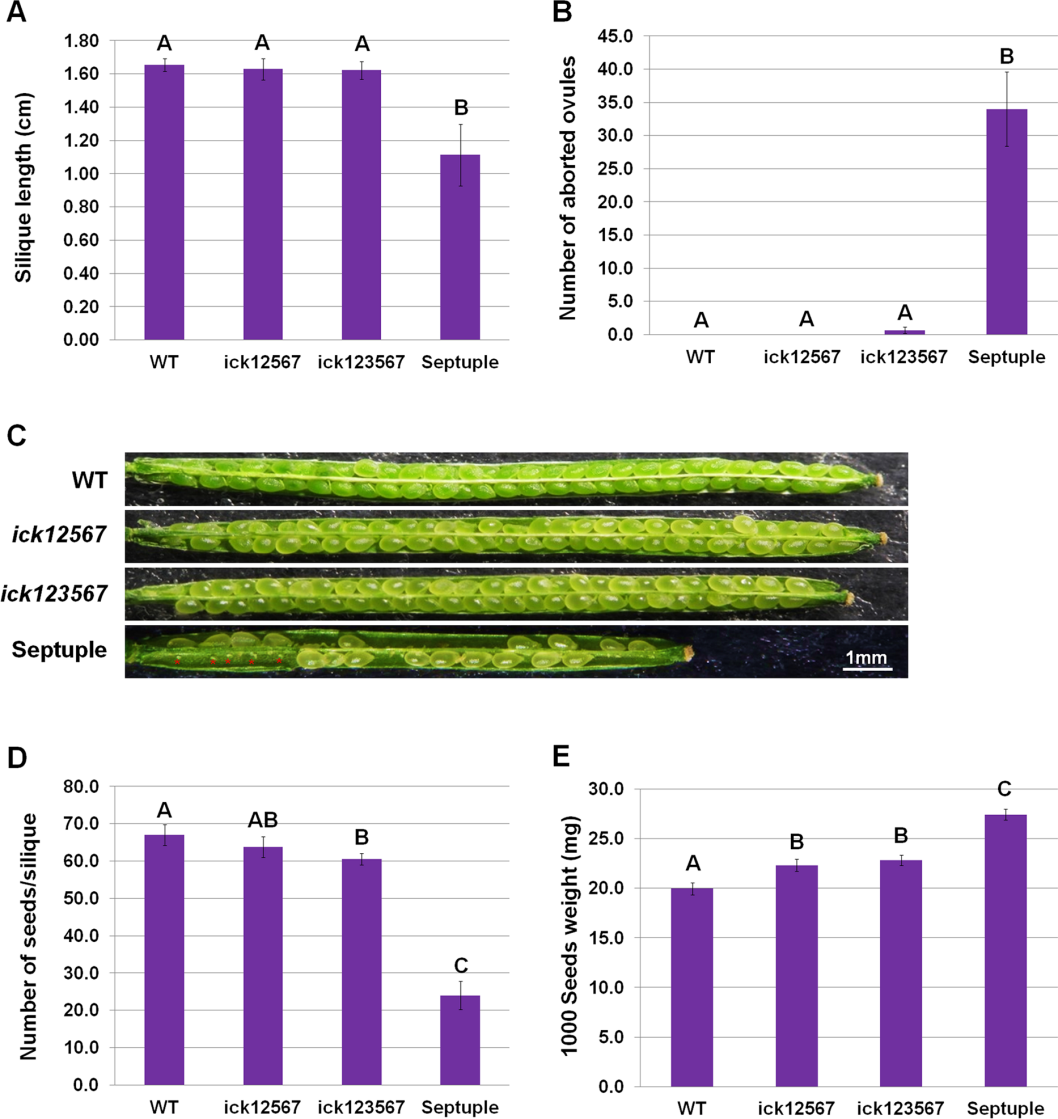
Silique and seed development in WT, *ick12567*, *ick123567* and septuple mutants. **(A)** Silique length. The average and standard deviation are shown for the length of fully extended siliques (4 plants per line with 10 siliques from each plant measured). (**B**) Number of aborted ovules per silique. Fully extended siliques were opened and aborted ovules counted under a dissecting microscope (4 plants with 6 siliques from each plant counted). The averages and standard deviations are shown. (**C**) Opened siliques showing silique length and aborted ovules of the septuple mutant. (**D**) Number of seeds per silique (4 plants per line with 6 siliques from each plant). The averages and standard deviations are shown. (**E**) Seed weight. Three different seed lots were used. For each lot, seeds were harvested from four plants in one pot and one thousand seeds were counted. The averages and standard deviations are shown. Data in (**A, B, D, E**) were analyzed using one-way ANOVA and post-hoc Tukey test, and significant differences are indicated by different letters (upper case) at p<0.01 level.

Compared with the wild type (WT) and quintuple *ick12567* mutant [26], the septuple mutant seedlings were slightly larger (S4A and S4B Fig). They had slightly larger first two pairs of leaves but the difference diminished in the 3^rd^ and 4^th^ pairs of leaves (S4C Fig). They had longer leaf blade (S4D Fig) and a slightly higher leaf length/width ratio (S4E Fig) for the 2^nd^ to 4^th^ pairs of leaves. These phenotypes were generally similar to, although slightly stronger than those of the quintuple mutant [26]. Interestingly, compared with the WT, quintuple and hextuple mutants, the septuple mutant had much shorter siliques (Fig 1A), a high frequency of aborted ovules (about 57%) (Fig 1B and 1C) and a reduced number of seeds per silique (Fig 1D). Previously, it was observed that the *ick12567* quintuple mutant had heavier seeds and more cells in cotyledons than the WT [26]. The septuple had even heavier seeds than the quintuple mutant (Fig 1E). Since the septuple mutant had a much reduced number of seeds per silique while the quintuple had a similar number compared to the WT (Fig 1D) [26], the increased seed weight in the septuple mutant could be due both to the direct effect of *ICK*/*KRP* inactivation on cell proliferation and the effect of reduced seed number per silique. The high frequency of aborted ovules in the septuple mutant suggested a defect in gametogenesis or early embryo development. To determine the parental contribution, reciprocal crosses were made between the WT and septuple mutant. The short silique phenotype was observed only when the septuple mutant was used as the female parent (S5 Fig), suggesting that a defect in female gametophyte development of the septuple mutant was responsible for the ovule abortion phenotype. Further, we analyzed pollen and observed that 90% of mutant pollen were normal with two sperm nuclei (n = 1151), compared to 98.7% in the WT (n = 1098), confirming that the mutant pollen grains were mostly normal (S1 Table).

### Development of multiple embryo sacs in the septuple *ick* mutant

**Fig 2:**
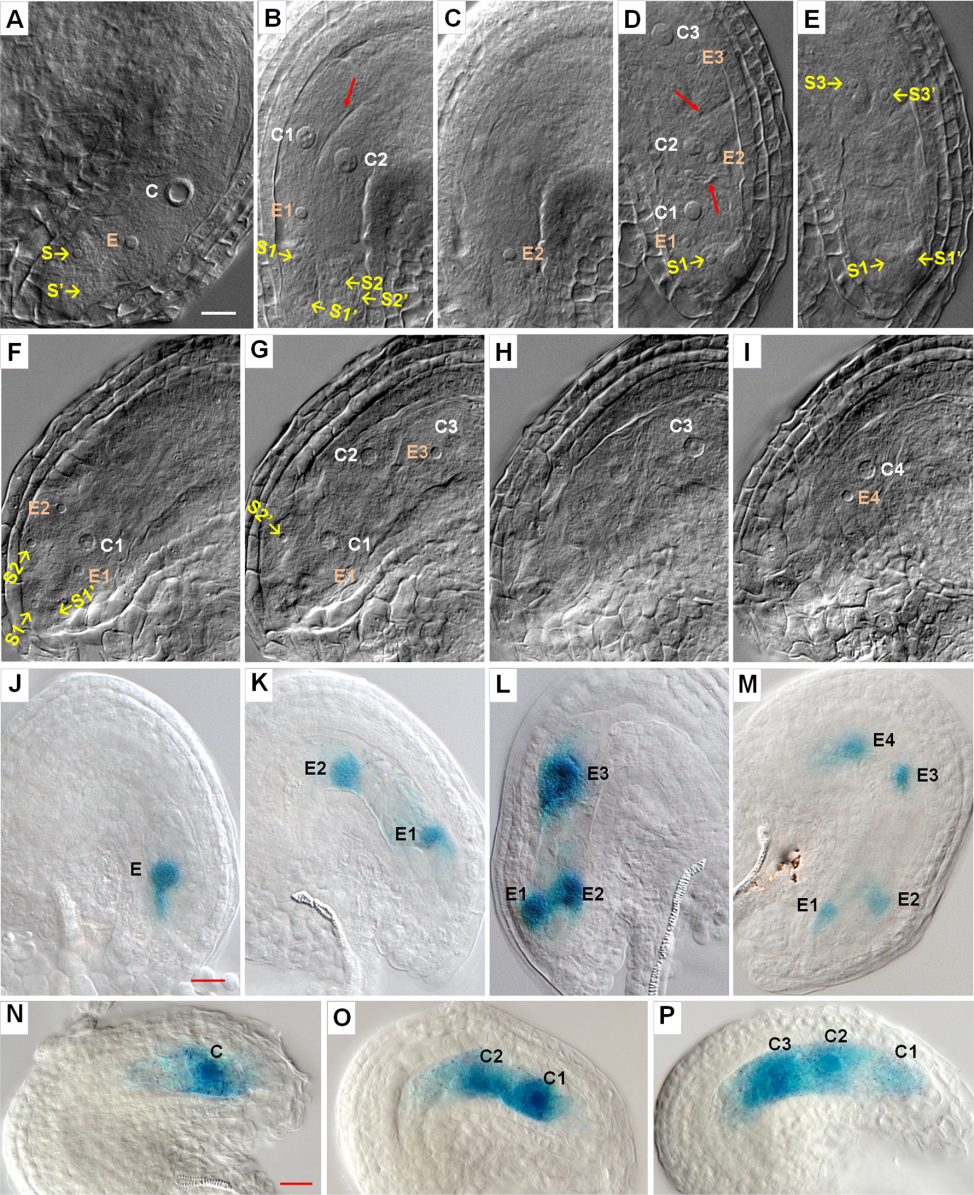
Embryo sac development in WT and *ick* septuple mutant. (**A—I**) DIC observation of embryo sac. Ovules from flowers just before opening were prepared and observed under a microscope with DIC optics. (**A**) A typical WT embryo sac showing secondary (central, C), egg (E) and synergid (S) nuclei. (**B—C**) Two optical sections of a septuple mutant ovule showing the embryo sac area with two sets of secondary (C), egg (E) and synergid (S) nuclei. The gamete and synergid nuclei are arranged in a configuration similar to that in the WT and the boundary between the two sets of gametes is clearly visible (arrow in **B**), indicating the presence of two embryo sacs in the ovule. (**D—E**) Two optical sections of a mutant ovule with three sets of secondary (C), egg (E) and synergid (S) nuclei. (**F—I**) Four optical sections of a mutant ovule with four set of secondary (C), egg (E) and synergid (S) nuclei. The synergids for the 3^rd^ and 4^th^ sets could not be easily observed. Different sets of gametes in the mutant are indicated by numbers “1”, “2”, “3” and “4”, respectively. The two syngergid nuclei of the same pair are differentiated with or without an “′”, e.g. “S1, S1′, S2, and S2′” referring to two pairs of synergid nuclei. (**J—M**) Ovules from the septuple mutant carrying a *pEC1*.*1*::*GUS* marker for the egg cell were stained for GUS. The GUS staining shows the presence of one to four egg cells in these ovules. (**N—P**) Ovules from the septuple mutant carrying a *pFIS2*::*GUS* marker for the central cell were stained for GUS. The GUS staining shows the presence of one to three central cells in these ovules. Scale bars in (**A**), (**J**), and (**N**) are for (**A—I**), (**J—M**), and (**N—P**) respectively, and all equal 10 μm.

To understand the reason for the ovule abortion phenotype in the septuple mutant, we examined embryo sac development. In the mature WT Arabidopsis mature embryo sac (FG7), the egg, central and two synergid cells, which could be easily recognized by their perspective nuclei, were arranged in a particular configuration (Fig 2A). Surprisingly, 47% of the septuple mutant embryo sacs (n = 186) did not have any recognizable egg, central cell (secondary) and synergid nuclei as observed in a mature WT embryo sac. On the other hand, about 46% of the ovules (93 out of 204 ovules surveyed), which contained gamete nuclei, had extra nuclei. In those ovules, the nuclei could usually be recognized as two, three or four sets of egg, secondary and synergid nuclei (Fig 2B to 2I and S2 Table). The egg and secondary nuclei of the same set were usually close to each other (e.g. Fig 2D). Within each set there was only one central and egg cell. Often clear boundaries between different sets of cells could be seen and distinguished (Fig 2B). These observations suggest the formation of multiple embryo sacs in the ovules of the septuple mutant, with each sac containing a set of egg and central cells. To further identify the gametes, we used a *pEC1*.*1*::*GUS* marker for egg cells [29] and *pFIS2*::*GUS* marker for central cells [30]. GUS staining showed the presence of multiple egg and central cells in the mutant ovules (Fig 2J to 2P), confirming the observations made with DIC microscopy.

To further confirm that the phenotypes are due to inactivation of *ICK*s, we performed complementation experiments. Re-introducing either an *ICK4* (S6 and S7 Figs) or *ICK7* genomic fragment (S8 and S9 Figs) was sufficient to rescue the septuple mutant. These results show that the observed phenotypes were due to specific inactivation of all *ICK*s, instead of a non-target gene(s). Since the difference between *ick123567* and the septuple mutant was the addition of the *ick4* mutant, we further investigated whether the phenotypes in the septuple mutant were due to any non-specific effect conferred by the *ick4* mutant. Thus, we obtained and analyzed the triple *ick467* and hextuple *ick123467* mutants along with WT, *ick4* single mutant and septuple mutant for *ick* gene expression, silique length, number of seeds and aborted ovules (S10 Fig). There seemed to be a trend of mild and gradual reductions in the silique length and number from the single to *ick123467* mutant (S11 Fig), similar to what was observed for *ick123567* mutant (Fig 1). However, the most dramatic changes were observed in the septuple mutant compared to the hextuple and lower order of mutants, indicating that the observed seed setting and ovule abortion phenotypes in the septuple mutant were specifically due to the inactivation of all seven *ICK* genes.

### Multiple megaspore mother cells and functional megaspores in the *ick* septuple mutant

**Fig 3:**
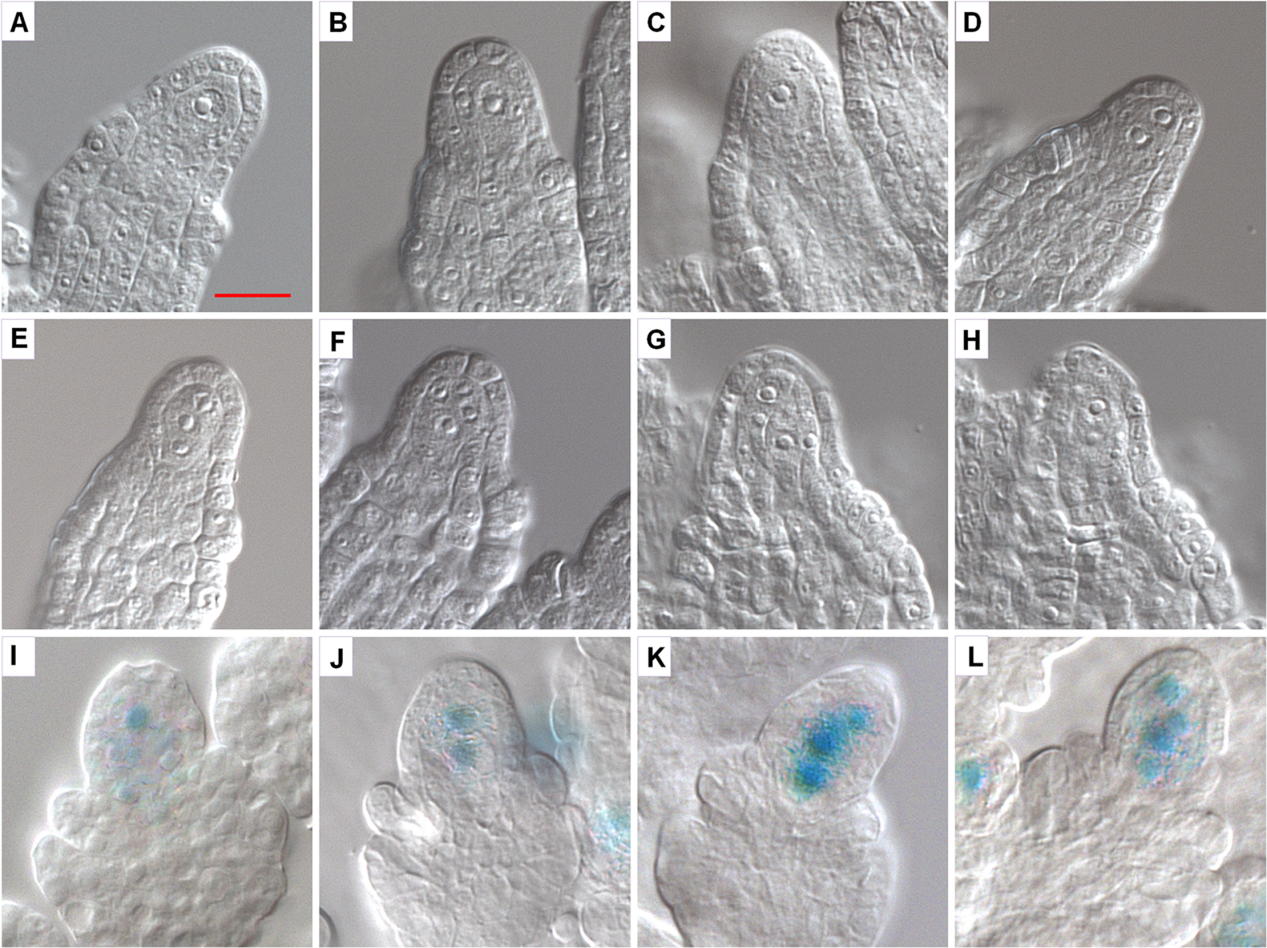
Development of megaspore mother cell and expression of the *pKNU-GUS* reporter in WT and septuple ovules. Developing ovules at stage 2-II were observed with DIC microscopy for development of megaspore mother cell (**A—H**) and ovules at around stage 2-III were analyzed for the expression of the *pKNU-nlsGUS* reporter, which is strongly expressed in MMC (**I—L**). (**A—B**) WT ovules. Most of WT ovules had one enlarged cell (**A**) and about 5% of ovules had two enlarged cells (**B**). (**C—H**) Septuple mutant ovules with one (**C**), two (**D**), three (**E**), four (**F**) and five (**G, H**) enlarged cells (**G** and **H** show two focal planes of the same ovule). (**I–J**) Strong *pKNU-nlsGUS* reporter expression relative to the surrounding cells indicates the presence of one (**I**), two (**J**), three (**K**) and four (**L**) MMCs in the mutant ovules. Scale bar in (**A**) is for all images and equals 10 μm.

The multiple embryo sacs observed in the septuple mutant could be due to multiple megaspore mother cells (MMCs) or multiple functional megaspores (FMs). To determine whether multiple MMCs were formed in the mutant, developing ovules at stage 2-II (when the inner integument is being initiated according to [31]) were surveyed using DIC (differential interference contrast) microscopy. In about 95% of WT ovules, there was only one MMC, while two enlarged MMC-like cells were observed in about 5% WT ovules (Fig 3A and 3B and S12A Fig). In the mutant ovules at the similar stage, about 19% of the ovules had one MMC, while majority of them had two to four, and in rare cases five, MMC-like cells (Fig 3C to 3H and S12A Fig). We further showed that a *pKNU-nlsGUS* marker which is known to be preferentially expressed in MMC [32] was expressed in the MMC-like cells in the mutant ovules (Fig 3I to 3L). Together, these results clearly demonstrate that multiple MMCs are formed in majority of the mutant ovules.

**Fig 4:**
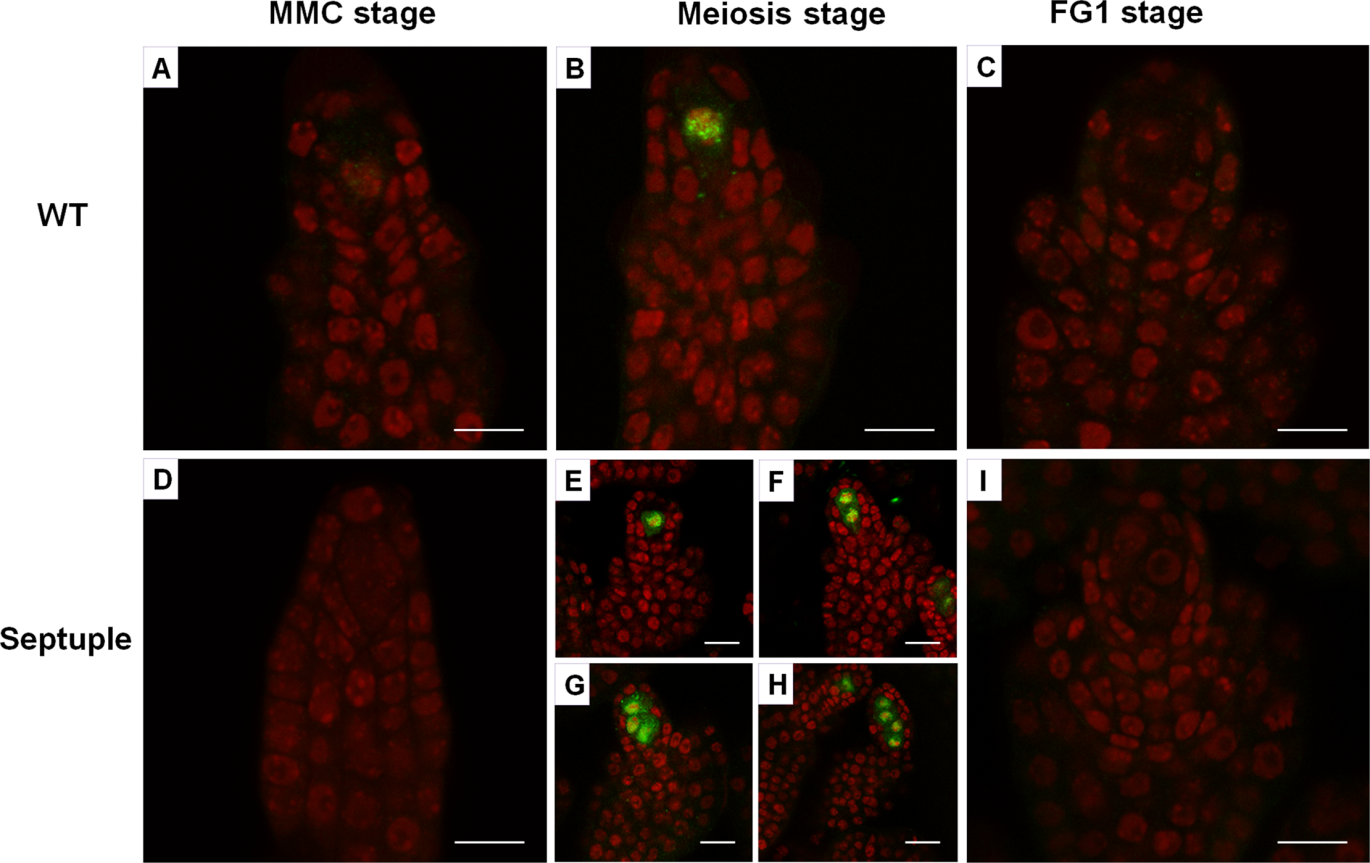
Identification of megaspore mother cells (MMCs) in meiosis by DMC1 immunostaining. Developing ovules were prepared and immunostained with an antibody against DMC1, which is specifically expressed during meiosis. (**A—C**) WT ovules at MMC (**A**), meiosis (**B**) and FG1 (**C**) stages. (**D—I**) Mutant ovules at MMC (**D**), meiosis (**E—H**) and FG1 (**I**) stages. One to four cells in the mutant ovules could be stained with DMC1 (**E—H**). Scale bars equal 5 μm.

To determine whether the multiple MMCs in the *ick* septuple mutant ovule are capable of entering meiosis, we performed immunostaining using antibodies against DMC1 and ASY1 proteins which have been shown to be specifically expressed during meiosis [33,34]. In WT ovules, only one MMC underwent meiosis, as indicated by the expression of DMC1 protein in one cell during meiosis and DMC1 protein was absent before or after meiosis (Fig 4A to 4C). On the other hand, in majority of the mutant ovules, two to four MMCs showed DMC1 protein expression (Fig 4F to 4H), with the frequencies for 2, 3, and 4 DMC1-expressing cells being around 67%, 15% and 3% respectively (S12B Fig). Similar results were obtained by immunostaining of another meiosis-expressing protein ASY1 (S13 Fig). These data showed that the multiple MMCs in the mutant ovule are able to enter meiosis, seemly at the same time.

**Fig 5:**
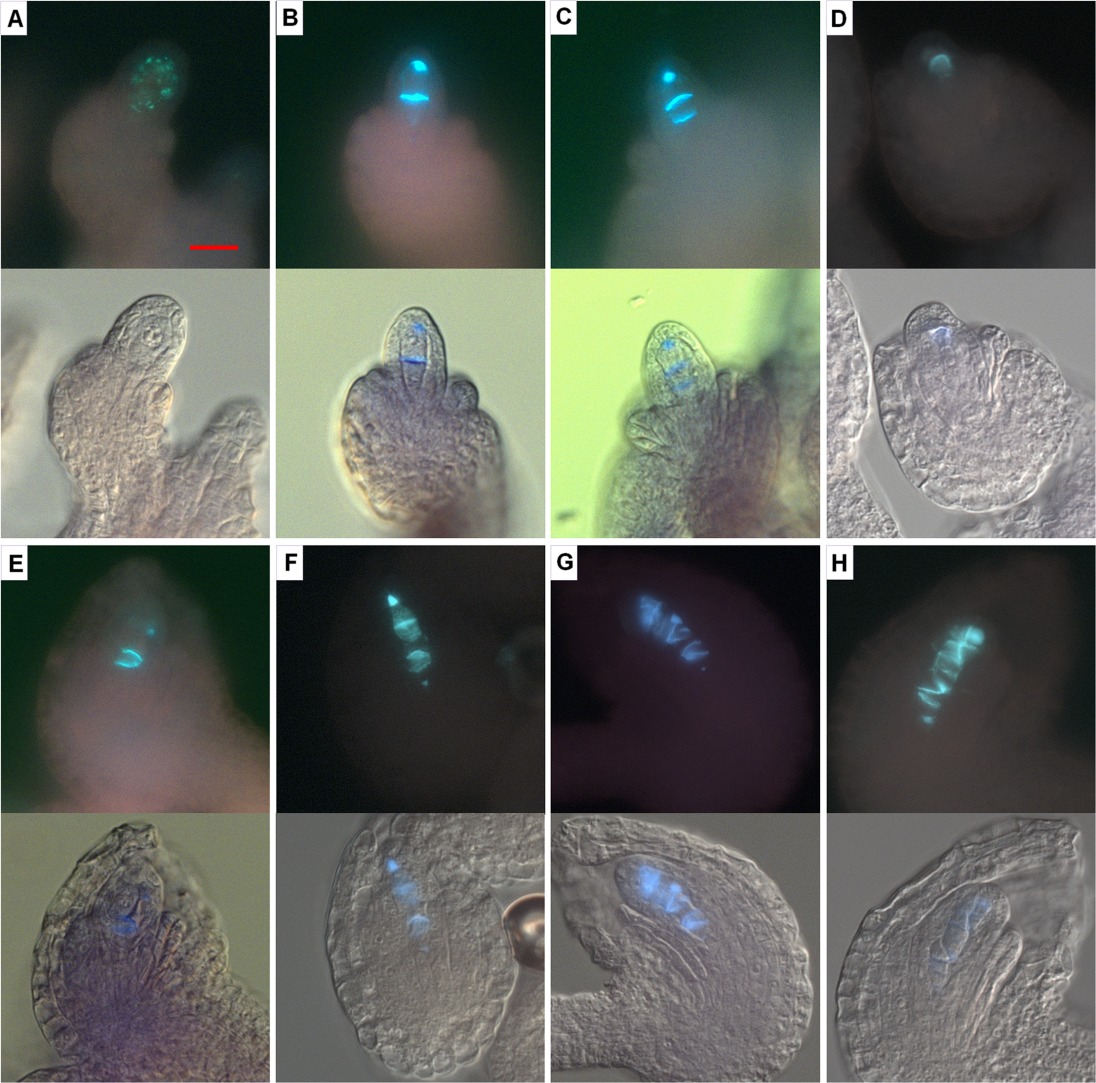
Analysis of callose deposition patterns in WT and septuple mutant ovules. Ovules were stained for callose with aniline blue. For each ovule, the callose fluorescence image is shown at the top and the DIC image with superimposed callose staining at the bottom. (**A—D**) WT ovules at different stages. (**A**) Before meiosis, there was weak and punctate callose deposition surrounding the MMC. (**B**) Callose deposition after the first division of meiosis showing a bright callose band (disc) at the place of newly formed cell plate. (**C**) Typical callose deposition pattern at the end of meiosis usually with two prominent calluse bands (discs) and also focused accumulation of callose at the micropylar end. One of the callose bands came from the first division and one from the second of the division by the nucleus close to the chalazal end. This pattern indicates the result of meiosis by one MMC. (**D**) At functional megaspore stage, the callose deposition was much reduced. (**E—H**) Septuple mutant ovules showing callose deposition from one (**E**), two (**F**), three (**G**) and likely four (**H**) MMCs. Scale bar in (**A**) is for all images and equals 10 μm.

During meiosis, callose is usually deposited at the newly synthesized cell plate following the first and second divisions, and can be detected by aniline blue staining. In the WT ovule, before meiosis, weak and punctate callose deposition was visible in the cell wall surrounding MMC (Fig 5A). Following the first division, a strong callose disk or band was present in the newly formed cell plate as well as strong callose deposition pointed at the micropylar end of the nucellus (Fig 5B). Following the second division, a new callose band below the first callose band was observed as a result of division by the daughter nucleus on the chalazal side. However little or no callose was observed for the expected cell plate from the division by the daughter nucleus close to the micropylar end (between the first callose band and the pointed callose deposition at micropylar end). As a result, the WT ovule at this stage had a typical callose pattern consisted of a middle band (usually strong) from the first meiotic division, a second callose band on the chalazal side and pointed callose deposition at the micropylar end, as shown in Fig 5C. If callose was present, the MMC in the WT ovule at different stages (before meiosis, after the first division and after the second division) could thus be distinguished by the callose deposition pattern. Based on the callose patterns, we therefore estimated the number of MMCs that have completed meiosis (Fig 5E to 5H; S12C Fig). In the septuple mutant, about 17% of ovules had one such typical callose deposition pattern from one MMC (Fig 5E), while about 61% ovules had two such callose patterns, and 22% of ovules had three or more such callose patterns (Fig 5F to 5H). These data clearly demonstrate that the multiple MMCs in the septuple mutant could complete meiosis.

**Fig 6:**
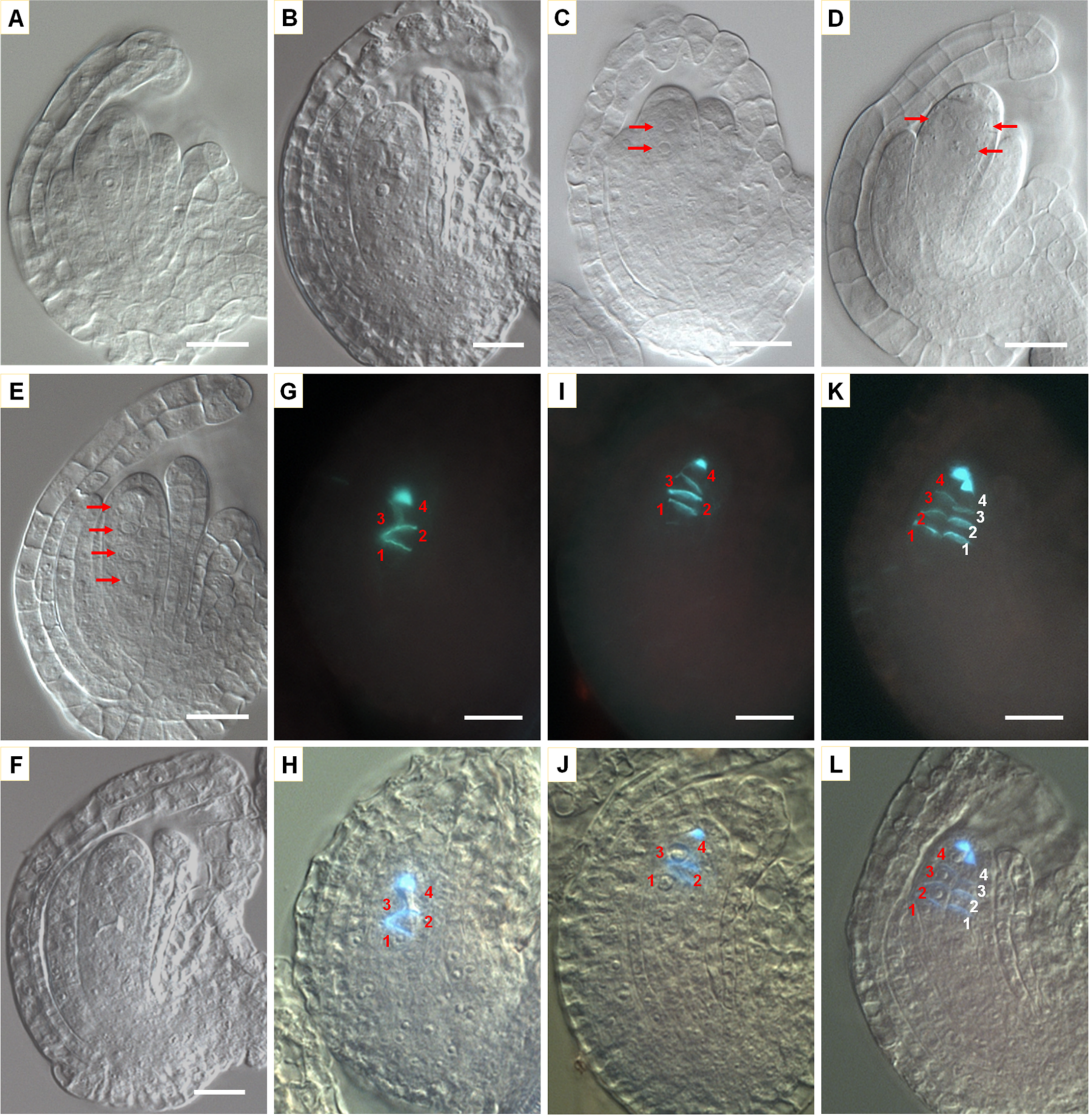
Functional megaspore (FM) development in WT and *ick* septuple mutant. (**A—F**) Ovules at functional megaspore stage and observed under a DIC microscope. (**A**) WT ovule with a typical FM. (**B–E**) *ick* septuple ovules with 1–4 FMs. A typical FM was present in (**B**). In (**C**), two pairs of nuclei appear to be derived from the second meiotic divisions. The two megaspores at the micropylar end are degenerating while the two close to the chalazal end were surviving. In (**D**), three megaspores of similar size and morphology were surviving, while in (**E**), four megaspores with different sizes and appearances were surviving. (**F**) No typical FM was observed in the ovule. (**G—L**) Callose staining and DIC images were obtained for the same mutant ovules at functional megaspore stage to determine which megaspore survived. The positions of megaspores from each MMC are indicated with a number, with position 1 indicating the megaspore closest to the chalazal end. (**G, I, K**) show callose staining while (**H, J, M**) show the corresponding DIC images with superimposed callose staining. (**G, H**) show that one MMC finished meiosis and two megaspores were surviving. (**I, J**) also show the result from one MMC, but megaspores at positions 1 and 3 (counting from the chalazal end) were surviving. Note that there are three callose bands plus the pointed callus deposition at the micropylar end. (**K, L**) indicate that two MMCs completed meiosis (**K**). While four megaspores derived from the MMC at one side were all surviving, no megaspore from the other MMC appeared to be surviving. Arrows in (**C–E**) indicate FMs, numbers in (**G–L**) indicate the megaspore positions counted from the chalazal end, and scale bars equal 10 μm.

In Arabidopsis as in most other plants, the MMC undergoes meiosis and produces four megaspores and only one megaspore close to the chalazal end becomes the FM while the other three degenerate [35]. We examined ovules at FM stage. In the WT ovule, of the four megaspores, the one close to the chalazal end survives to become the FM (Fig 6A) and no WT ovule with two FMs was observed. The FM in WT ovule was recognizable by a large nucleus and often visible nucleus. The nuclei of the degenerative megaspores were either very small or not visible (Fig 6A). In ovules of the septuple mutant, one to several FMs were observed (Fig 6B to 6E, and S3 Table). When there was one FM, it appeared similar to the FM in the WT (Fig 6B). For ovules with multiple FMs, in some ovules the multiple FMs appeared similar to each other (Fig 6C and 6D), while in other ovules they differed in size and morphology (Fig 6E). Furthermore, in some mutant ovules there was no recognizable FM except for some small nuclei, indicating that the megaspores in those ovules underwent degeneration or had degenerated (Fig 6F). A quantitative survey further revealed that about 29% of mutant ovules did not have an observable FM, 30% had one FM, and the remaining had two or more FMs (S3 Table).

The occurrence of multiple MMCs in the mutant ovules raises a question regarding whether each of the multiple FMs was derived from one MMC as in the WT. It was difficult to determine the origin of each FM under the DIC microscope since the boundaries among the FMs from multiple MMCs were often not clear. However, sometimes the multiple FMs in an ovule were arranged in a way indicating that they were likely derived from one MMC. For instance, in Fig 6C, four megaspores were likely produced by the same MMC, with the two pairs of megaspores produced through the two second meiotic divisions. The two megaspores at the micropylar end seemed degenerating based on the much smaller size while the two megaspores close to the chalazal end appeared surviving. Similarly, in Fig 6D, three megaspores that appeared to be from one MMC resembled the functional megaspore in the WT.

To further address this issue, we obtained the callose and DIC images of the same ovules. Since a recognizable callose pattern was left by one MMC following meiosis (Fig 5C), when both callose deposition and FMs could be observed, we could determine whether the multiple FMs were originated from one or different MMCs. The callose staining in Fig 6G indicates the completion of meiosis by one MMC and overlaying of the callose image with the DIC image revealed that the two megaspores close to the chalazal end were surviving (Fig 6H). In the WT, cell plate from the division by the dyad cell at the micropylar side was usually not observed (Fig 5C). In contrast, this callose band was often clearly observed in septuple mutant ovules resulting in a callose pattern consisted of three callose bands plus the pointed callose deposition at the micropylar end (Fig 6I). In this ovule, the first and third megaspores from the chalazal end were surviving (Fig 6J). The callose pattern in Fig 6K indicates clearly that two MMCs had completed meiosis. While four megaspores derived from the MMC at one side were all surviving, no megaspore from the other MMC appeared to be surviving (Fig 6L). These results indicate that megaspores from different MMCs could have very different fates and multiple megaspores derived from one MMC could survive in the septuple mutant.

To exclude the possibility that the presence of multiple MMCs might have affected the selection and polarity of surviving megaspores, we examined megaspore survival when there was only one MMC in the mutant ovule. Ovules with one MMC could be determined based on the callose staining pattern as well as DIC image since following meiosis ovules with multiple MMCs had more complex callose patterns and cellular arrangements than ovules with one MMC (Fig 5E to 5H). Our analysis showed that in the WT, only the megaspore closest to the chalazal end survives without an exception (n = 120 ovules), while for the mutant ovules with one MMC, both the number and position of surviving megaspores varied (S14 Fig). The frequencies for 0, 1, 2, 3 and 4 surviving megaspores were 13%, 42%, 37%, 4% and 4% respectively (7, 22, 19, 2 and 2 ovules out of 52 surveyed). Further, the frequency of surviving megaspores for each of the four positions was between 17% to 30%, with the megaspore at positions 1 and 4 (counting from the chalazal to micropylar end) having a slightly higher frequency (Table 1).

**Table 1:** Frequency of surviving megaspores at each of the four positions in *ick* septuple mutant ovules with one MMC.

Line	Position of surviving megaspore	Frequency
WT	1st	120 (100%)
Septuple	1st	24 (30%)
	2nd	17 (21%)
	3rd	13 (16%)
	4th	20 (25%)
	none survived	7 (9%)

The mutant ovules with one MMC were identified after meiosis based on callose staining with aniline blue and DIC microscopy. In the WT, only the megaspore closest to the chalazal end survives. The surviving megaspores in the mutant are grouped based on their positions. The position numbers indicate the 1^st^ to 4^th^ positions from the chalazal to micropylar end.

**Fig 7:**
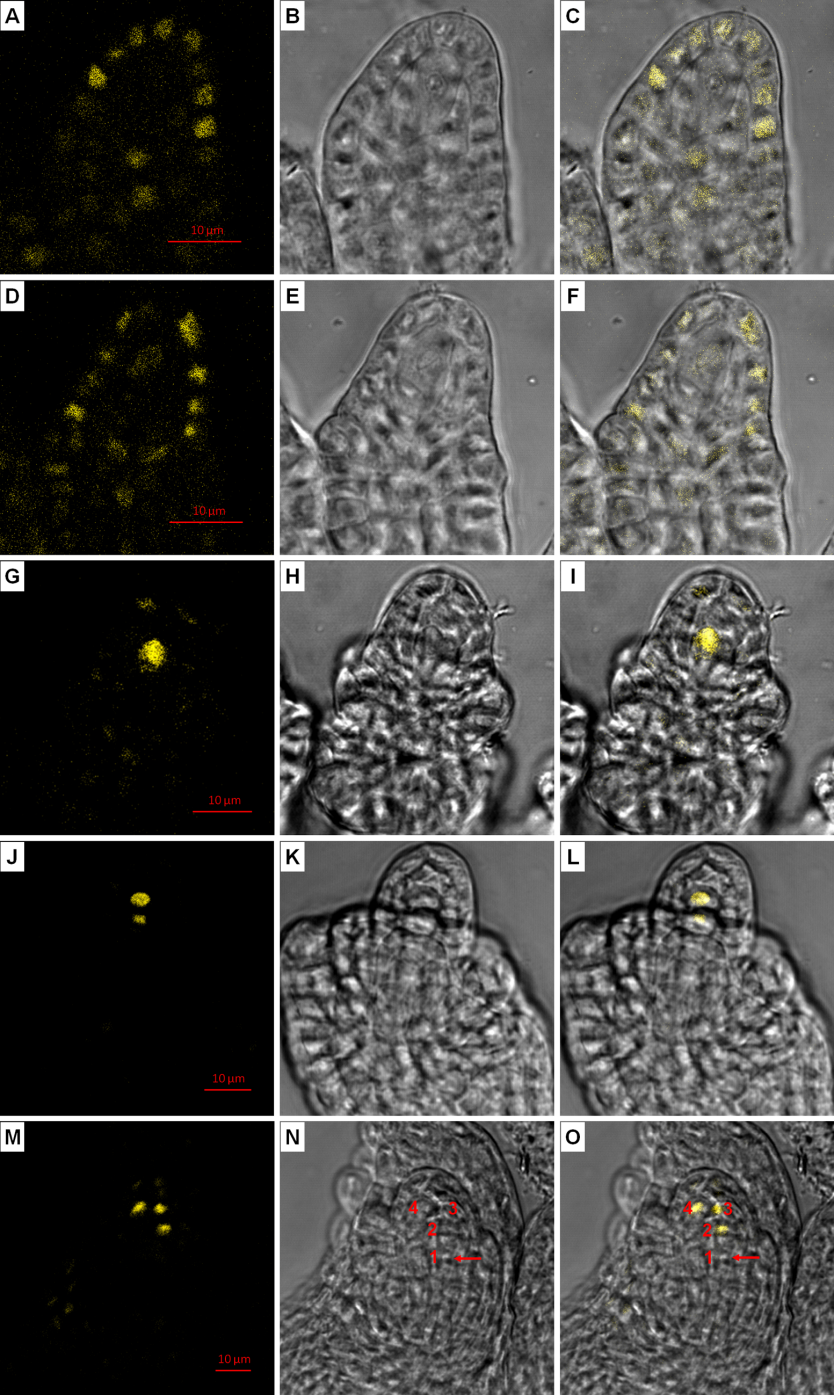
ICK4-YFP expression during megasporogenesis. Developing ovules of ICK4-YFP transgenic line (WT background) were observed under a confocal microscopy. (**A, D, G, J, M**) show confocal images, (**B, E, H, K, N**) show bright field images, and **(C, F, I, L, O**) show the overlays of the confocal and bright field images. (**A—C**) A stage 2-I ovule. (**D—F**) A stage 2-II ovule. (**G—I**) A stage 2-III ovule. (**J—L**) A stage 2-IV ovule at the end of meiosis I. (**M—O**) A stage 3-I ovule after meiosis. The numbers in (**N, O**) indicate the positions of megaspores counting from the chalazal to micropylar end. The arrow in (**N, O**) points to the nucleus of functional megaspore. The scale bars in (**A**), (**D**), (**G**), (**J**) and (**M**) are for (**A—C**), (**D—F**), (**G—I**), (**J—L**) and (**M—O**) respectively.

These results clearly suggest a critical role of ICKs in restricting the number of MMCs and also in the selective degeneration of megaspores. To gain an understanding on ICK expression during ovule development, we fused ICK4 with YFP (yellow fluorescence protein). We used an ICK4 lacking the C-terminal 29 amino acids so that it should not have the CDK inhibitory activity [6] and would not affect normal megasporogenesis. As reported previously [36], ICK4-YFP was localized in the nucleus (Fig 7). In stage 2-I and earlier stage ovules, ICK4-YFP was preferentially expressed in L1 layer and cells surrounding MMC, but not in MMC or progenitor cell (Fig 7A to 7C). Its expression was gradually shifted to MMC during its formation, visible in stage 2-II ovules (Fig 7D to 7F) and very strong in stage 2-III ovules (Fig 7G to 7I). Following meiosis I, ICK4-YFP was present more in the nucleus at the micropylar side (Fig 7J to 7L). Strikingly, after meiosis II, it was strongly present in the degenerative megaspores and consistently absent in the FM (Fig 7M to 7O). These observations reveal a dynamic pattern of ICK4 protein expression: in cells surrounding MMC in early-stage ovules, gradually becoming concentrated in MMC before meiosis, in the micropylar nucleus following meiosis I, and then strongly present in degenerative megaspores.

Further at FG2 stage during female gametogenesis, the WT embryo sac had one typical pair of nuclei (S15A Fig) while the mutant ovules showed two to four pairs often with clear boundaries between them (S15B to S15D Fig), indicating that multiple FMs could develop further in gametogenesis in the same ovule.

### Double embryos and multiple endosperm compartments in the *ick* septuple mutant

**Fig 8:**
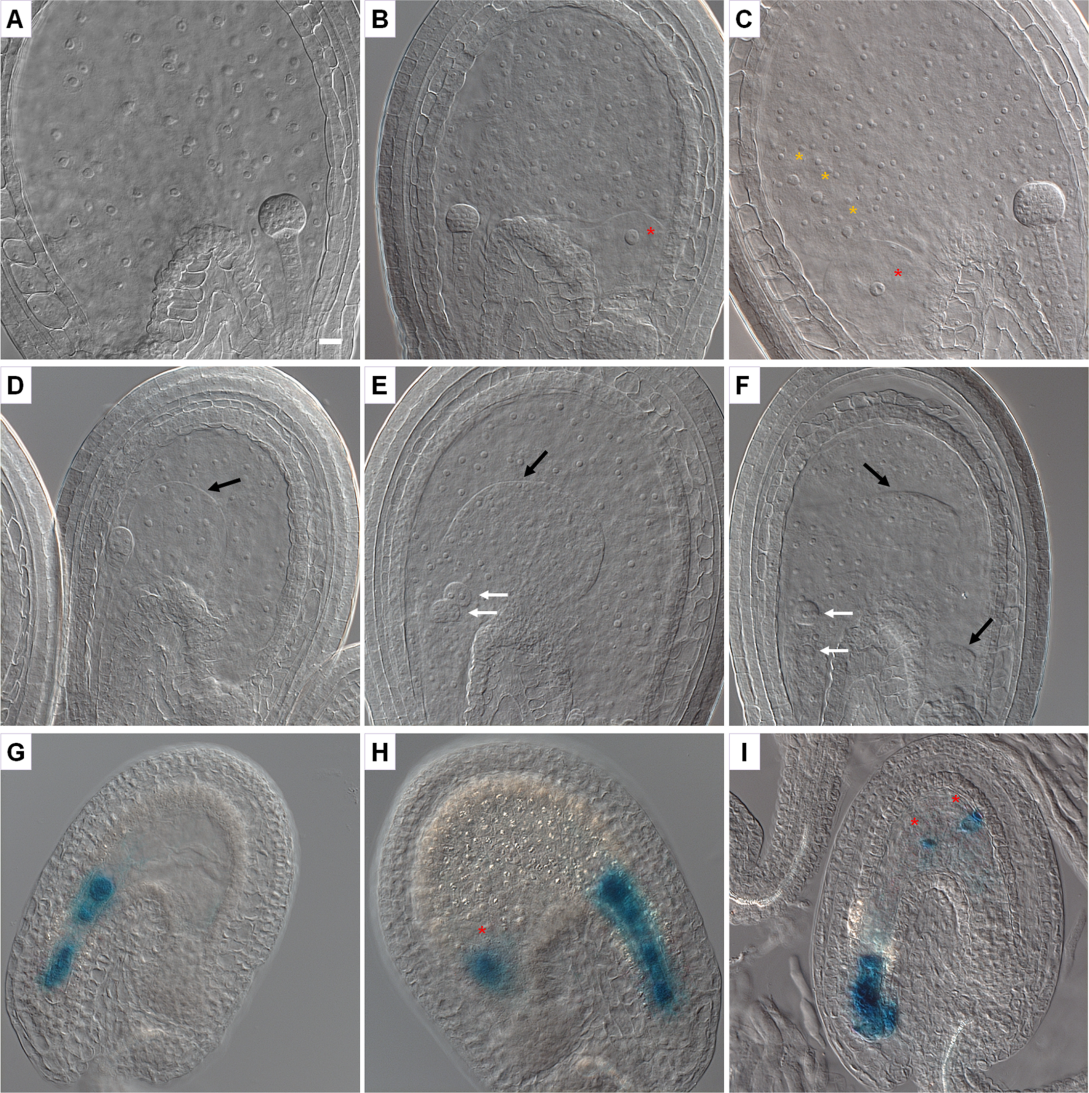
Embryo and endosperm development in WT and *ick* septuple mutant. Three days after flowers opened, the young developing seeds were prepared for DIC observations or stained for GUS first and then prepared for observations. (**A**) A typical WT embryo sac with a globular embryo and endosperm nuclei of uniform morphology distributed throughout the embryo sac. (**B—F**) Embryo and endosperm development in mutant ovules. (**B**) In addition to the typical embryo and endosperm, a separate compartment is seen to contain a secondary nucleus. (**C**) In the chalazal area, there are also a few nuclei like the secondary nucleus (yellow star signs) in the endosperm as well as a secondary nucleus in a separate embryo sac (red star signs). (**D**) There is one embryo, and two separate compartments of endosperm. (**E**) There are two embryos, and two separate compartments of endosperm. (**F**) There are two embryos, and three separate compartments of endosperm. (**G—I**) Embryo and egg cells identified by *pEC1*.*1*::*GUS* expression in the septuple mutant. (**G**) A young embryo showing GUS staining. (**H**) In addition to the developing embryo, strong GUS staining is seen at the chalazal end of the embryo sac where a separate sac is often present. (**I**) In addition to the developing embryo, GUS staining is present at two other loci indicating the presence of extra egg cells. White arrows indicate embryos and black arrows indicate the boundaries between two separate compartments of endosperm. Stars indicate extra egg cells or secondary nuclei. Scale bar in (**A**) is for (**A—I**) and equals 10 μm.

**Fig 9:**
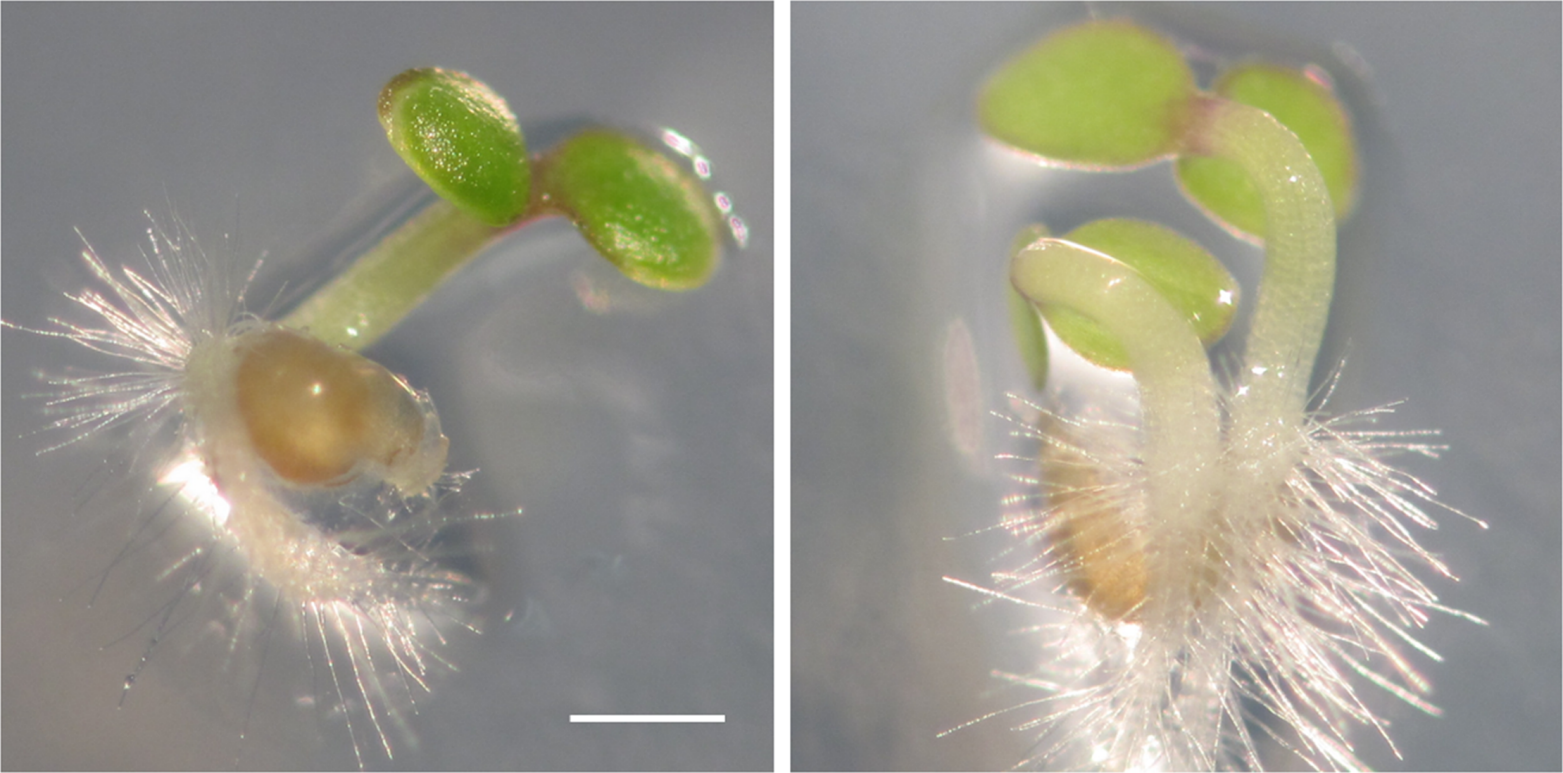
Occurrence of twin seedlings in the *ick* septuple mutant. A typical 4-day Arabidopsis seedling (left) and twin mutant seedlings (right). Scale bar equals to 1 mm.

We further investigated the embryo development. Three days after flowering, the WT ovules had one embryo with the endosperm nuclei of uniform morphology distributed throughout the embryo sac (Fig 8A), whereas in the mutant ovules different types of nuclei and interestingly two embryos were observed (Fig 8B to 8F). In the mutant ovules with one embryo, additional nuclei, distinct from the endosperm nuclei, resembling and presumably derived from the unfertilized secondary nuclei, were found in different locations (Fig 8B and 8C). Often, in addition to one endosperm, a separate sac with a secondary nucleus was observed at the chalazal end, indicating that an unfertilized embryo sac with the membrane still intact had been pushed to the chalaza by the fertilized and expanding embryo sac (Fig 8B and 8C). Some ovules had one embryo but two separate endosperm compartments (Fig 8D), suggesting the possible development of one endosperm compartment without an embryo. About 2.6% mutant ovules had two embryos and typically two separate endosperms with a clear boundary (Fig 8E), and in rare cases three separate endosperm compartments were observed (Fig 8F). An analysis was performed to determine the frequency of ovules with more than one endosperm compartment as well as extra nuclei resembling the secondary nucleus (S4 Table). All WT ovules (n = 168) had one embryo and one endosperm compartment with uniform nuclei without any secondary nucleus, while about 26% mutant ovules had two endosperm compartments (S4 Table). In addition, extra secondary nuclei were found in one location (about 15%) or two locations (about 3.2%) in the mutant ovules. GUS staining of ovules after fertilization revealed the expression of *pEC1*.*1*::*GUS* marker in developing embryo (Fig 8G), and also confirmed the presence of egg nucleus in the embryo sac at the chalaza (Fig 8H) or in the developing endosperm (Fig 8I). Consistent with the development of double embryos, twin seedlings (Fig 9) were observed in about 2.1% mutant seeds (58 out of 2760 seeds), while none was observed in the WT seeds (S5 Table).

## Discussion

In plants, the germline cells are not set aside early during embryogenesis as in animals, but differentiate much later in the life cycle from somatic cells. The female gametophyte development is consisted of two phases: megasporogenesis and megagametogenesis. In megasporogenesis, one somatic cell in the nucellus of a young ovule enlarges, changes in morphology and further differentiates into a megaspore mother cell (MMC), which is defined by its ability and commitment to undergo meiosis [37,38]. Thus, formation of an MMC involves a differentiation process as well as the change of fate from mitosis to meiosis. The MMC undergoes meiosis producing four megaspores, and in majority of angiosperms only one of the four megaspores survives to become the FM [35,39]. In megagametogenesis, the FM develops into an embryo sac or the female gametophyte following three rounds of mitosis. Cytological observations have suggested that a group of nucellar cells is competent to differentiate into MMCs while the first MMC formed suppresses the formation of additional MMCs [40], and also that the non-functional megaspores need to be suppressed or all megaspores would develop [39]. However, little is known regarding the molecular mechanisms for suppressing other nucellar cells from differentiating into MMCs or the survival of non-functional megaspores following meiosis.

Several studies have suggested a role in restricting the differentiation of multiple MMCs by a type of receptor-like kinases and a small interacting protein. In rice, *MULTIPLE SPOROCYTE* (*MSP1*) encodes a leucine-rich repeat receptor-like kinase and the *msp1* mutant had excess megasporocytes and microsporocytes [41]. Further, the TAPETUM *DETERMINANT1* (*TPD1*) encodes a small protein that interacts with MSP1 and down-regulation of *TPD1A* in rice resulted in excess megasporocytes but not microsporocytes [42]. Excess megasporocytes and microsporocytes were also observed in the maize mutant of *Multiple Archesporial Cells 1* (*MAC1*) which is an ortholog of rice *TDL1A* and Arabidopsis *TPD1* [43,44]. In Arabidopsis, the corresponding genes are *Excess Microsporocytes1* (*EMS1*) or *EXTRA SPOROGENOUS CELLS* (*EXS*) encoding the leucine-rich repeat receptor-like kinase [45,46] and *TPD1* encoding the interacting protein [47]. The Arabidopsis *ems1*, *exs*, and *tpd1* mutants all had excess microsporocytes, but without any megasporocyte phenotype [45,46,48]. These results indicate that the MSP1/TPD1 pathway is critical for restricting the development of excess megasporocytes and microsporocytes in rice and maize, but the related pathway in Arabidopsis does not have a similar role in restricting the development of excess megasporocytes. In addition, certain ARGONAUTE proteins (AGOs) of the small RNA pathways are also implicated in suppressing the differentiation of megasporocytes. In the Arabidopsis *ago9* mutant, 37%– 48% of the premeiotic ovules had multiple enlarged MMC-like cells, however only one of them was capable of entering meiosis [49]. The *ago4* and *ago6* single mutants also showed similar phenotypes; however the *ago4 ago9* double mutant was found to suppress the frequency of ovules with excess MMC precursor cells [50]. These results seem to suggest that *AGO* genes function in a complex way in preventing the differentiation of multiple megasporocyte precursor cells prior to meiosis. However, the specific mechanisms involved remain unknown. The multiple MMC phenotypes in the septuple mutant are different from those of the *ago9* and related mutants in that usually only one MMC of the *ago9* mutant is able to undergo meiosis although excess MMC-like cells are formed [49].

The multiple MMCs in the *ick* septuple mutant could be differentiated from multiple nucellar cells. In such a case, ICK/KRPs in the WT plants would function to restrict the additional nucellar cells from developing into MMCs. Alternatively, the multiple MMCs in the septuple mutant might be due to extra rounds of mitotic divisions by a single MMC after its differentiation. While our manuscript was under preparation, a study was published and showed that triple *ICK*/*KRP* mutants as well as *rbr1* mutant also display multiple MMC and embryo sac phenotypes [51]. Based on their results, Zhao et al. conclude that ICK/KRPs and RBR1 restrict MMC from entering mitosis in the WT while in the mutants the MMC undergoes extra mitotic divisions resulting in the formation of supernumerary MMCs. The preferential transcript expression of *ICK4*/*KRP6* and *ICK5*/*KRP7* in MMC [17,51] and the strong ICK4-YFP protein expression observed in this study support this conclusion. However, this role of ICK/KRP proteins in preventing MMC from entering mitosis also raises interesting new questions. First, at which stage do the mitotic divisions by the MMC occur? The mitotic divisions by enlarged MMCs should occur often in the septuple mutant ovules and be relatively easy to observe. However, we did not observe the apparent signs of mitotic divisions by enlarged MMCs in the septuple mutant. It is likely that the extra cell divisions might have occurred at an early stage of ovule development by the MMC or its progenitor cell. Second, more intriguingly, if the high level of ICKs in the MMC is to prevent it from entering mitosis and since ICK/KRP proteins target mainly CDKA and D-type cyclins [8,9], the fact that meiosis seems uninhibited suggests that a different CDK or CDK complex might be responsible for initiating meiosis in the MMC.

Although the multiple MMC phenotype was similarly observed in our study and by Zhao et al. [51], our study provides additional novel findings with implications for the functions of ICKs. First, we provided independent lines of evidence for an important role of ICKs in the degeneration and selection of megaspores (see discussion below). Second, in the septuple mutant we observed double embryos with separate endosperm compartments and also twin seedlings, demonstrating that two female gametophytes in the mutant ovule could develop simultaneously and complete the double fertilization independently. Third, we used mainly the septuple mutant compared to triple mutants used by Zhao et al. The observation that septuple mutant has stronger and additional phenotypes (e.g. double embryos and seedlings) than other lower orders of mutants suggests functional redundancy of all ICKs in this process. Consistent with this conclusion, we observed that under our experimental conditions the frequency of ovules with multiple MMCs was about 17% for the *ick123467* and *ick123567* hextuple mutants, compared to over 80% for the septuple mutant.

Although the basic molecular functions of the major cell cycle regulators have been relatively well established, how these cell cycle regulators are specifically wired into various developmental processes is not clearly understood. Previously, some cell cycle regulators have been found to be important for certain aspects of germline cell specification and gametogenesis. In the Arabidopsis *CDKA;1* mutant, the second mitosis in pollen is inhibited resulting in one-sperm pollen [52]. In the heterozygous mutant of RBR1, a negative cell cycle regular through repressing E2F transcription factors, the embryo sacs have excess nuclei due to extra rounds of cell divisions [53]. In addition, FBL17, an F-box protein, has been shown to target several ICK/KRP proteins for degradation and the heterozygous mutant of *fbl17* displays a one-sperm pollen phenotype similar to that of the *cdka;1* mutant [15,16]. Interestingly, mature pollen grains in the *ick* septuple mutant were mostly normal with 90% of them containing two sperm nuclei, compared to 98.7% in the WT. The 8.7% difference was due to mutant pollen with no or one sperm nucleus (5.4% and 3.3% respectively), suggesting that the *ICK* knockout only has a relatively small effect on pollen development. Thus, the present results suggest the ICKs play more critical roles in megasporogenesis and female gametophyte development.

Since multiple MMCs in the septuple mutant could complete meiosis, an interesting question is whether each MMC in the mutant produces one FM as in the WT ovule. We observed that in some mutant ovules, two or more megaspores from the same MMC could survive, while in others all megaspores seemed to undergo degeneration. In this regard, it has been shown that overexpression of an Arabidopsis cell wall protein AGP18 resulted in the survival of multiple megaspores acquiring an FM identity, but those megaspores failed to develop further [54]. Our results clearly suggest that ICK/KRPs are involved in the degradation of megaspores and selection of the FM. First, when *ICK*s are inactivated, more than one megaspore could survive and the surviving megaspores could be at any position. Second, ICK4-YFP was specifically and strongly present in degenerative megaspores but absent in FM. Based on these results, we propose that the expression or presence of ICK4 and presumably other ICKs in the degenerative megaspores promotes their degeneration, while its absence allows FM to develop further. Consistent with this notion, it has been shown that overexpression of ICK/KRPs promotes the degeneration of pollen and female gametes [17,23] as well as trichomes [24]. Further, the mammalian KIP/CIP CDK inhibitors are known to have proapoptotic roles [reviewed in [55]], and the role of ICKs in promoting megaspore degeneration is consistent with the known roles of CDK inhibitors in cell death in animals. It will be interesting to determine the underlying mechanism for the absence of ICK4 expression in the FM. Considering that ICKs are highly controlled post-translationally [56], it is likely that ICK4 and presumably other ICKs are removed from FM following meiosis. In addition, our results also show that the ICK4-YFP fusion protein (and likely with a different reporter) can serve as a useful marker for identifying degenerative megaspores.

Our results point to dual functions for ICK/KRPs in restricting the number of MMC and in the degeneration of megaspores. The MMC differentiation and degeneration of non-functional megaspores are closely connected events, both spatially and temporarily. The process of non-functional megaspore degeneration likely has initiated before the completion of meiosis, since frequently after the first meiotic division the cell close to the micropylar end degenerates without completing the second meiotic division [57]. Indeed, we observed that ICK4-YFP had stronger expression in the nucleus at the micropylar end following meiosis I (Fig 7J to 7L) and further the cell plate from the second division by the daughter cell at the micropylar end was usually not observed in WT ovules by callose staining, but frequently observed in mutant ovules. These results suggest that the second meiotic division at the micropylar end is often incomplete in WT, and when *ICK*s are inactivated the daughter cell at the micropylar end could divide more normally and the resulting megaspores could survive, compared to their destined degeneration in the WT.

In angiosperms, only one embryo is formed in each ovule following double fertilization, in which the egg and central cells are fertilized by two sperm nuclei of a single pollen grain. The synergids play the role of pollen guidance and sperm release. During double fertilization, usually only one pollen tube reaches the embryo sac and releases the two sperm nuclei with the receptive synergid degenerating at the same time [58]. After fertilization, a block is established to prevent more pollen tubes from entering the embryo sac [59]. In the septuple mutant, double embryos were found in about 2.6% ovules and twin seedlings in about 2.1% seeds. The double embryos from sexual reproduction may be formed in some mutant plants due to two different embryo sacs [60], the embryogenic transformation of suspensor cells [61], or splitting of the same zygote [62]. Double embryo formation has also been reported in the Arabidopsis *amp1* mutant which has supernumerary egg cells at the expense of synergids, however the double embryos do not develop further due to the absence of endosperm [63]. The formation of double embryos with separate endosperm compartments in the septuple *ick* mutant indicates clearly that the double embryos are a result of two separate double fertilization events occurring to two embryo sacs. Such an interesting phenotype is rarely seen in other mutants of known genes, suggesting a possible function of *ICK/KRP*s in fertilization or embryo development.

We observed a trend of mild and gradual decreases in silique length and seed number from lower to hextuple mutants indicating some dosage effect (Fig 1 and S11 Fig). However, the dramatic phenotypes in ovule abortion and seed setting were only observed in the septuple mutant indicating that the strong phenotypic effects in the septuple mutants require inactivation of most or all seven *ICK/KR*Ps. The fact that in vast majority of flowering plants only one MMC is formed per ovule and only one megaspore is selected to become FM implies a positive selection pressure during evolution for these developmental features to ensure that only one embryo would develop per seed. There is some evidence to suggest that seedlings from double embryos may be less fit than seedlings from single embryos [64]. The wide range of abnormal outcomes in the *ick* septuple mutant from the absence of gametes to multiple embryo sacs and to the development of double embryos suggests that the formation of one MMC, selection of one FM and its development into one embryo sac in normal plants is through a highly regulated developmental pathway, in which ICK/KRPs play important roles. Thus, one reason for the increased number of *ICK/KRP* genes in seed plants [4] may be to ensure strong redundancy and stability of this important regulatory system.

## Materials and methods

### Plant materials and growth conditions

*Arabidopsis thaliana* ecotype ‘‘Columbia” and its mutant lines were grown in a growth room or chamber (20 °C constant, 16/8 h day/night photoperiod with a fluence rate of 90 ± 10 μmoles/m^2^/min). The quintuple *ick* mutant and the single T-DNA mutants from which the quintuple mutant was created have been described in [26]. The *ick3* (SALK_053533) and *ick4* (Sail_548_B03) T-DNA mutants were obtained from the Arabidopsis Biological Resource Center (Ohio State University). These lines are in the Columbia (Col-0) ecotype background. Crosses were made as described in S2 Fig to obtain the double *ick34*, triple *ick467*, hextuple *ick123467* and *ick123567*, and septuple mutants.

### Genotyping and gene expression analyses

Arabidopsis genomic DNA was extracted for genotyping analysis by PCR. Genotyping of the T-DNA insertion lines was performed as described[26]. For gene expression analysis, total RNA was isolated using TRIzol Reagent (Invitrogen) according to manufacturer’s instructions. For RT-PCR, first-strand cDNA was synthesized using the Invitrogen ThermoScript RT-PCR system from 1.5 μg of total leaf RNA.

### Phenotyping of the mutant lines

For seedling biomass analysis, seeds of WT, *ick12567* and septuple mutants were planted in soil in the plant growth room. For each line, three pots each having five plants were used in the analysis. At 21 days after planting, seedlings were collected from each pot and weighed immediately for the fresh weight. Experiments were repeated at four different locations in the growth room. The relative fresh weight for each mutant (fresh weight of mutant/fresh weight of WT) was obtained.

For morphological phenotyping, the 1^st^, 2^nd^, 3^rd^ and 4^th^ pairs of true leaves from seedlings at the indicated plant ages were separated, placed on a flat surface and photographed with a digital camera. The leaf sizes, leaf length and width were measured from the obtained images using ImageJ software (http://rsbweb.nih.gov/ij/index.html). The leaf length/width ratio for each line was obtained from its length and width data. For seed weight analysis, mutants and control plants were grown side by side to minimize the variation in growth conditions. Seeds were collected on the basis of one pot each having four plants, and three seed lots (each from the four plants in one pot) were used, with 1000 seeds from each seed lot counted and weighed. Three different batches of seeds from plants grown at different locations of the plant growth room were analyzed. For the analyses of silique and seed development, ten fully extended siliques (6^th^ -15^th^ from the bottom) from four plants per line were measured. Six measured siliques per plant were opened using a pairs of sharp tweezers, and counted for the number of the seeds and aborted ovules under a Zeiss dissecting microscope. For statistical analysis, one-way ANOVA and post-hoc Tukey test (using the software SPSS) were performed to determine the significance between lines.

### Complementation experiments

A 3786-bp *ICK4* genomic region (consisted of 2040 bp before ATG, the coding region and 717 bp of the region after the STOP codon) was amplified from Arabidopsis genomic DNA with *Pfu* DNA polymerase as a fragment containing *BamH*I and *Sac*I sites with the primers HW1001 (5’-gactggatcccttgacatagagttttctaca) and HW1002 (5’-gactgagctcattactactccgcataggc). An *ICK7* genomic region (consisted of 1335 bp before ATG, the coding region and 838 bp of the region after the STOP) was similarly amplified with the primers HW1003 (5’-cagtggatccggtctctttacgaatatctta) and HW1004 (5’-cagtgagctcgatatgtagtgagtgggtac). The fragments were cut with *BamH*I and *Sac*I enzymes, and cloned into *pCambia1300* that has the hygromycin resistant marker for plant selection (http://www.cambia.org/daisy/cambia/585.html). The constructs were used to transform the septuple mutant. Transformants were selected on the 1/2 MS plates containing 40 μg/ml hygromycin and 300 μg/ml timentin, and grown in a tissue culture chamber. T2 plants were genotyped by PCR and plants with confirmed genotypes were used for analyzing gene expression and phenotypes.

### Light microscopy

For observation of female gametophyte development, emasculated flowers or developing buds at specific stages were used. Sample preparations and DIC (differential interference contrast) microscopic observations were performed as described [65]. Photographs were taken using a Leica DFC450C digital microscope camera. For observations of endosperm and embryo development, the siliques at 3 days after fertilization (flowering) were collected, opened along both sides of the pistil replum using a pair of sharp tweezers to expose ovules, fixed for 1 hour, and washed as described above. The ovules were detached from the placenta, placed in 60 μl clearing solution (chloral hydrate: water: glycerol (8:2:1)) in a 0.5 ml tube and incubated at room temperature for 2 hrs to clear. The ovules were pipetted onto a piece of glass slide, covered with a coverslip and observed under a Leica DM2500 microscope.

Aniline blue staining was based on a procedure described [66] with modifications. Briefly, the gynoecia were collected, cut along the carpel using a scalpel or a pair of super sharp tweezers, and fixed in FAA solution overnight. They were then hydrated in 25%, 15% ethanol and water sequentially each for 10 min, placed in 0.1% aniline blue in a microtube and vacuumed infiltrated. After incubation in dark for 4 hours, they were washed twice with PBS for 20 min each. Ovules were picked up and mounted on a glass slide. Fluorescence was observed under a Leica DM2500 microscope using a 340–380 nm bandpass excitation filter and 425 nm long-pass emission filter.

### Immunolocalization of DMC1 and ASY1 proteins

Immunolocalization of DMC1 and ASY1 proteins in young ovules was performed as described [67]. Rabbit anti-DMC1 and anti-ASY1 antibodies [68] were used at 1:100. The images were captured using a Leica SP5 confocal microscope.

### Cell identity markers and ICK4-YFP expression

For preparing GUS report constructs, first a *Hind*III-*EcoR*I fragment containing a promoter::GUS reporter was cut from a pBI121-based construct previously prepared in our lab and cloned into *pCamiba1300* (resulting in S113-D2). The 1819-bp *FIS2* [30] and 1716-bp *EC1*.*1* [69] promoter regions were amplified, with the primers of **5’-c**agtaagcttcgcatctttttttcttctttc and **5’-**cagt ggatccctgcttgattaatctataagc for *FIS2* promoter, and the primers of **5’-c**agtaagcttgttgctggaacctgttcc and **5’-**cagtggatcctctcaacagattgataaggtc for ec1.1 promoter. The *Hind*III-*BamH*I fragments was cloned into the modified *pCambia1300*, resulting *ProFIS2*::*GUS* and *ProEC1*.*1*::*GUS*. For the *pKNU-nlsGUS* reporter [32], a 2420-bp genomic region containing the promoter region and the coding region for the first 138 amino acids were amplified with following primers containing *Hind*III and *Sal*I sites respectively: 5’-cagtaagctttggtagatttgttctgtgca and 5’-cagtgtcgacttgttaccggataatgcaaaag. The amplified fragment was cloned into a GUS reporter vector S274D10, a modified *pCamiba1300* containing GUS reporter with an SV40 nuclear localization signal. The constructs were introduced into the septuple mutant. Transformants were selected as described in the above. A number of transformants were transferred to and grown in soil. Histochemical GUS staining was performed as described [70] with minor modifications. Ovules were fixed with 3.7% formaldehyde in GUS staining buffer basal (100mM PO_4_^3-^ (pH 7.0), 2 mM K_3_(Fe(CN)_6_), 2 mM K_4_(Fe(CN)_6_), 10 mM Na_2_EDTA (pH 8.0), 0.08% Triton X-100 and 10 mM Na2EDTA) for 10 min, washed in GUS staining buffer basal twice for 5 min each, stained in GUS staining buffer (0.5 μg/ml X-gluc), vacuumed for 1 min under 600 mm Hg vacuum and incubated at 37°C overnight. After fixation in FAA solution for 10 min, the samples were washed with 25% ethanol, 10% ethanol and then water for 5–10 min each, mounted in the clearing solution and examined under the microscope.

To determine ICK4 protein expression, an *ICK4* genomic fragment, spanning the 4100-bp sequence upstream of ATG and 167 amino acid region downstream of ATG, was amplified with the primers: 5’-cagtctgcagaattagttgtccatagttgtg and 5’-cagtgtcgactcttgactctctagctccg. After the restriction digest, the genomic fragment was cloned into a YFP vector and translationally fused to the N-terminus of YFP. The construct was used to transform Arabidopsis WT plants. ICK4-YFP expression was analyzed in the initial transformants and progeny plants.

## Supporting information

S1 Fig Genotyping and RT-PCR of *ick3* and *ick4* T-DNA insertion mutants. (**A**) Genotyping of *ick3* (SALK_053533) and *ick4* (Sail_548_B03) single mutants with WT as a control. For both genes, a pair of gene-specific primers was used for amplifying the full-length WT coding sequence of *ICK3* or *ICK4* while a T-DNA left border primer and a gene specific primer were used to amplify a fragment of the T-DNA insert allele. (**B**) Analysis of *ICK3* transcript in WT (left lane) and *ick3* single mutant (right lane) by RT-PCR. cDNA synthesized from total RNA and gene-specific primers were used for amplifying the full-length *ICK3* coding sequence (upper row), and the reference *actin* sequence (lower row). (**C**) Analysis of *ICK4* transcript in WT (left lane) and *ick4* single mutant (right lane) by RT-PCR. cDNA synthesized from total RNA and gene-specific primers were used for amplifying the full-length *ICK4* coding sequence (upper row), and the reference *actin* sequence (lower row).
(PDF)

S2 Fig Crosses used to produce various *ick* mutants from *ICK* single mutants and *ick12567* mutant. (PDF)

S3 Fig Genotyping and RT-PCR of *ick* septuple mutant. (**A**) Gene-specific primers for *ICK1*, *ICK2*, *ICK3*, *ICK4*, *ICK5*, *ICK6* and *ICK7* were used to confirm the genotype of *ICK* septuple mutant with WT genomic DNA as a control. (**B**) Analysis of *ICK* transcripts in the WT (first lane) and septuple mutant (second lane), with WT genomic DNA as a control (third lane). Gene-specific primers were used for amplifying the full-length sequences of *ICK1* to *ICK7*. *Actin* was used as a control (the last row).
(PDF)

S4 Fig Phenotyping of WT, *ick12567* and septuple mutant plants. (**A**) Seedlings of WT, *ick12567* and septuple mutants at 26 days after planting in soil. (**B**) Fresh seedling weight of 21-day-old WT, *ick12567* and septuple mutants. For each line, 3 pots each with 5 plants were used in the analysis. The averages and standard deviations are shown. (**C—E**) Leaf area (**C**), blade length (**D**) and leaf length/width ratio (**E**) of 21-day-old WT, *ick12567* and septuple mutant plants (6 plants from each line were used). The 1^st^, 2^nd^, 3^rd^ and 4^th^ pairs of true leaves of WT, *ick12567* and septuple mutant were separated, placed on a flat surface, and their photos were taken with a digital camera. The leaf area, leaf blade length and width were measured using ImageJ software. The leaf length/width ratio for each leaf was obtained from its length and width. The averages and standard errors are shown. Data in (**B—E**) were analyzed using one-way ANOVA and post-hoc Tukey test, and significant differences are indicated by different letters (lower case) at p<0.05 level.
(PDF)

S5 Fig Reciprocal crosses to determine the transmission of the short silique phenotype through the parents. Reciprocal crosses (indicated as female X male) were made between the WT and septuple mutant. The length of fully-elongated siliques was measured. In each treatment, four plants with 4 siliques for each plant were used. The averages and standard deviations are showed. Data were analyzed using one-way ANOVA and post-hoc Tukey test, and significant differences are indicated by different letters (upper case) at p<0.01 level.
(PDF)

S6 Fig Genotyping of WT, septuple mutant and complementation lines with a genomic *ICK4* fragment. An *ICK4* genomic fragment (including 2040 bp before ATG, 1028 bp of the coding region and 717 bp of the region after the STOP codon) was introduced into the septuple mutant. Multiple independent lines were identified that showed complementation of the silique length phenotypes. Two independent T2 lines are presented here. PCR was used to determine the genotypes of the WT, septuple mutant and complementation lines. For each gene, duplex PCR was performed to detect the WT allele (with gene-specific primers for the full-length coding region) and the T-DNA allele using (with a gene-specific primer and a left border primer of T-DNA). The complementation lines are the same as the septuple mutant except for the presence of the WT *ICK4* band.
(PDF)

S7 Fig RT-PCR and phenotyping of WT, septuple mutant and complementation lines with a genomic *ICK4* fragment. (**A**) *ICK4* transcript analysis. cDNAs synthesized from total RNAs were used to detect the *ICK4* transcripts in WT, septuple mutant and two complementation lines (T2 generation). *Actin (ACT8)* was amplified as a control. (**B**) Silique length of WT, septuple mutant and the two complementation lines. Four plants and in each plant the 6^th^ -15^th^ siliques from the bottom of the main florescence were measured. The averages and standard deviations are showed. (**C**) Opened siliques of WT, septuple mutant and two complementation lines showing the seed development. Scale bar = 1 mm. (**D**) Number of seeds per silique (the 6^th^ -15^th^ siliques per line in the main inflorescence from each plant). The averages and standard deviations are showed. (**E**) Number of aborted ovules per silique. Fully extended siliques (10 siliques for each line) were opened and aborted ovules counted under a dissecting microscope. The averages and standard deviations are shown. Data in (**B, D, E**) were analyzed using one-way ANOVA and post-hoc Tukey test, and significant differences are indicated by different letters (upper case) at p<0.01 level.
(PDF)

S8 Fig Genotyping of WT, septuple mutant and complementation lines with a genomic *ICK7* fragment. An *ICK7* genomic fragment was introduced into the septuple mutant. Many transformants (6/40) with normal silique length were observed and the analysis of two independent transformants (1243–4 and 1243–23) is shown here. PCR was used to determine the genotypes of the WT, septuple mutant and transformants with *ICK7* genomic fragment. For each gene, duplex PCR was performed to detect the WT allele (with gene-specific primers for the full-length coding region) and the T-DNA allele using (a gene-specific primer and a left border primer of T-DNA). The transformants are the same as the septuple mutant except for the presence of the WT *ICK7* band.
(PDF)

S9 Fig RT-PCR and phenotyping of WT, septuple mutant and transformants with *ICK7*. (**A**) *ICK7* transcript analysis. cDNAs synthesized from total RNAs were used to detect the *ICK7* transcripts in WT, septuple mutant and two transformants with *ICK7* genomic fragment. *Actin (ACT8)* was amplified as a control. (**B**) Silique length of WT, septuple mutant and the two transformants. Ten siliques (6^th^-15^th^) from the bottom of the main florescence were measured for each plant. The averages and standard deviations are showed. (**C**) Opened siliques of WT, septuple mutant and the two transformants showing the seed development. Scale bar = 1 mm. (**D**) Number of seeds per silique (8 siliques (7^th^-14^th^) in the main inflorescence from each plant). The averages and standard deviations are showed. (**E**) Number of aborted ovules per silique. Fully extended siliques (8 siliques for each plant) were opened and aborted ovules counted under a dissecting microscope. The averages and standard deviations are shown. Data in (**B, D, E**) were analyzed using one-way ANOVA and post-hoc Tukey test, and significant differences are indicated by different letters (upper case) at p<0.01 level.
(PDF)

S10 Fig Genotyping of WT, *ick4*, *ick467*, *ick123467* and septuple mutants. Gene-specific primers for *ICK1*, *ICK2*, *ICK3*, *ICK4*, *ICK5*, *ICK6* and *ICK7* genes, and a T-DNA left border primer were used to confirm the genotypes of WT, *ick4*, *ick467*, *ick123467* and septuple mutants.
(PDF)

S11 Fig RT-PCR and phenotyping of WT, *ick4*, *ick467*, *ick123467* and septuple mutants. (**A**) Analysis of *ICK* transcripts in the WT (the first lane), *ick4* (the second lane), *ick467* (the third lane), *ick123467* (the fourth lane) and septuple mutant (the fifth lane), with WT genomic DNA as a control (the last lane). Gene-specific primers were used for amplifying the full-length sequences of *ICK1* to *ICK7*. *Actin* was used as a control (last row). (**B**) Silique length. The average and standard deviation are shown for the length of fully extended siliques (4 plants per line with 8 siliques from each plant measured). (**C**) Opened siliques showing silique length and aborted ovules of WT, *ick4*, *ick467*, *ick123467* and septuple mutants. (**D**) Number of seeds per silique (4 plants per line with 6 siliques from each plant). The averages and standard deviations are shown. (**E**) Number of aborted ovules per silique. Fully extended siliques were opened and aborted ovules counted under a dissecting microscope (4 plants per line with 6 siliques from each plant). The averages and standard deviations are shown. Data in (**B, D, E**) were analyzed using one-way ANOVA and post-hoc Tukey test, and significant differences are indicated by different letters (upper case) at p<0.01 level.
(PDF)

S12 Fig Number of megaspore mother cells determined by DIC, DMC1 immunostaining and callose deposition patterns. (**A**) WT and septuple ovules at stage 2-II were surveyed under a DIC microscope and determined for the number of enlarged MMC-like cells prior to meiosis. Number of ovules counted: WT = 129 and mutant = 149. (**B**) Number of MMCs undergoing meiosis as revealed by immunostaining with an antibody against DMC1, a protein specifically expressed during meiosis, but not at MMC and FG1 stages (for images see Fig 4). Number of ovules counted: WT = 59 and mutant = 60. (**C**) Number of MMCs estimated based on the callose deposition following meiosis. The callose deposition could be attributed to one, two, three or more MMCs since a typical pattern is produced as the result of meiosis from one MMC. Number of ovules counted: WT = 127 and mutant = 227.
(PDF)

S13 Fig Identification of megaspore mother cells (MMCs) in meiosis by ASY1 immunostaining. Developing ovules were prepared and immunostained with an antibody against ASY1, which is specifically expressed during meiosis. (**A—C**) WT ovules at MMC (**A**), meiosis (**B**) and FG1 (**C**) stages. (**D—I**) Mutant ovules at MMC (**D**), meiosis (**E—H**) and FG1 (**I**) stages. One to four cells in the mutant ovules could be stained with DMC1 (**E—H**). Scale bars equal 5 μm.
(PDF)

S14 Fig Analysis of surviving megaspores in WT and septuple mutant ovules with one MMC. Ovules with a single MMC were identified based on callose staining with aniline blue and DIC microscopy. For each ovule, the callose fluorescence image is shown at the top and the DIC image at the bottom. The numbers in the images indicate the 1^st^ to 4^th^ megaspore position from the chalazal to micropylar end. (**A—B**) WT ovule with one megaspore at the most chalazal position surviving. (**C—L**) show mutant ovules. (**C—D**) Mutant ovule with one megaspore at the 4^th^ position surviving. (**E—F**) Two megaspores at the 1^st^ and 2^nd^ positions surviving. (**G—H**) Two megaspores at the 1^st^ and 4^th^ positions surviving. (**I—J**) Two megaspores at the 3^rd^ and 4^th^ positions surviving. (**K—L**) All four megaspores seemly surviving. Scale bar in (**A**) is for all images and equals 10 μm.
(PDF)

S15 Fig Early megagametogenesis in WT and *ick* septuple mutant. WT (**A**) and septuple (**B—D**) ovules at FG2 stage. The WT ovule had one pair of nuclei. The mutant ovules had two (**B**), three (**C**) and four (**D**) pairs of nuclei, with each pair likely derived from a functional megaspore. The two nuclei in each pair are close to each other and have similar appearance. The boundary between different pairs is often visible (**B, D**). Different numbers in (**B—D**) indicate different pairs of nuclei. Scale bar in (**A**) is for all images equals 10 μm.
(PDF)

S1 Table Analysis of mature pollen grains in WT and *ick* septuple mutant. Mature pollen grains from ten nascent flowers each line were collected and stained with DAPI. Vegetative and sperm cell nuclei were observed and counted under a fluorescence microscope (Leica DM2500) with a 63× (NA = 1.40) oil lens.
(PDF)

S2 Table Observation of female gametes in mature embryo sacs in the WT and *ick* septuple mutant. The flower buds just before opening were emasculated and left for 1 day. Ovules (from about 15 gynoecia for each line) were prepared and observed under a microscope with DIC optics. If one central cell (secondary) nucleus and one egg nucleus, usually close to each other, could be identified, they were considered as one set. The synergid nuclei were not always recognizable when there were three or four sets of gametes. For the mutant, a large portion (about 47% by a separate analysis) of embryo sac did not contain any observable secondary and egg nuclei, and were not included in this survey.
(PDF)

S3 Table Development of functional megaspore (FM) in ovules of the WT and *ick* septuple mutant. The ovules were checked and those at functional megaspore stage were included in the analysis. They were observed under a microscope with DIC optics. The presence and number of functional megaspores in ovules (from about 15 gynoecia) were counted.
(PDF)

S4 Table Analysis of endosperm development and presence of extra central cells after fertilization in the WT and *ick* septuple mutant. Three days after flowers opened, ovules were collected and prepared for DIC observation. Only the ovules containing at least one developing embryo were included (about 20 gynoecia from each line used). All the WT ovules had one endosperm compartment with uniform endosperm nuclei, while some mutant ovules had two endosperm compartments. Nuclei resembling the secondary nucleus were also present in the endosperm of the mutant. The secondary nucleus might have divided into several nuclei, which were recognizable against the background of endosperm nuclei (See Fig 8C). The nuclei that were close to each other physically and looked similar morphologically were considered to be derived from one original secondary nucleus. “+1CC”, “+2CC” and “+3CC” indicate the number of central cells that are inferred to based on the extra secondary nuclei present.
(PDF)

S5 Table Frequency of twin seedlings in the WT and *ick* septuple mutant. WT and septuple mutant seeds were plated on ½ MS plates. Four days after plating, seedlings were screened under a dissecting microscope for the occurrence of twin seedlings.
(PDF)
