# Supplementary material for: Arabidopsis ICK/KRP cyclin-dependent kinase inhibitors function to ensure the formation of one megaspore mother cell and one functional megaspore per ovule
Source: PLoS Genet. 2018 Mar 7;14(3):e1007230. doi: 10.1371/journal.pgen.1007230 (PMC5858843; doi:10.1371/journal.pgen.1007230)
Supplement: S1 Fig — (A) Genotyping of ick3 (SALK_053533) and ick4 (Sail_548_B03) single mutants with WT as a control. For both genes, a pair of gene-specific primers was used for amplifying the full-length WT coding sequence of ICK3 or ICK4 while a T-DNA left border primer and a gene specific primer were used to amplify a fragment of the T-DNA insert allele. (B) Analysis of ICK3 transcript in WT (left lane) and ick3 single mutant (right lane) by RT-PCR. cDNA synthesized from total RNA and gene-specific primers were used for amplifying the full-length ICK3 coding sequence (upper row), and the reference actin sequence (lower row). (C) Analysis of ICK4 transcript in WT (left lane) and ick4 single mutant (right lane) by RT-PCR. cDNA synthesized from total RNA and gene-specific primers were used for amplifying the full-length ICK4 coding sequence (upper row), and the reference actin sequence (lower row). (PDF) [file pgen.1007230.s001.pdf]

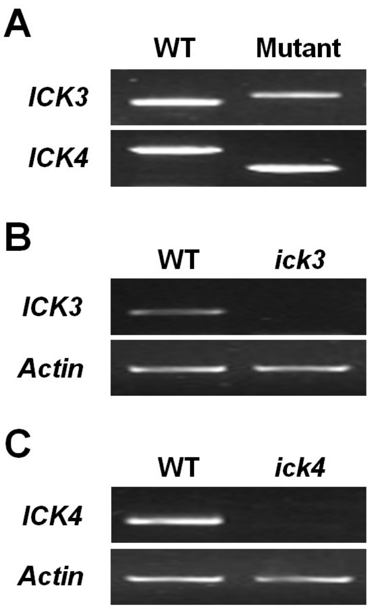


**Figure S1. Genotyping and RT-PCR of *ick3* and *ick4* T-DNA insertion mutants.**
(**A**) Genotyping of *ick3* (SALK_053533) and *ick4* (Sail_548_B03) single mutants with WT as a control. For both genes, a pair of gene-specific primers was used for amplifying the full-length WT coding sequence of *ICK3* or *ICK4* while a T-DNA left border primer and a gene specific primer were used to amplify a fragment of the T-DNA insert allele.
(**B**) Analysis of *ICK3* transcript in WT (left lane) and *ick3* single mutant (right lane) by RT-PCR. cDNA synthesized from total RNA and gene-specific primers were used for amplifying the full-length *ICK3* coding sequence (upper row), and the reference *actin* sequence (lower row).
(**C**) Analysis of *ICK4* transcript in WT (left lane) and *ick4* single mutant (right lane) by RT-PCR. cDNA synthesized from total RNA and gene-specific primers were used for amplifying the full-length *ICK4* coding sequence (upper row), and the reference *actin* sequence (lower row).
