# Supplementary figures and images for: Arabidopsis ICK/KRP cyclin-dependent kinase inhibitors function to ensure the formation of one megaspore mother cell and one functional megaspore per ovule
Source: PLoS Genet. 2018 Mar 7;14(3):e1007230. doi: 10.1371/journal.pgen.1007230 (PMC5858843; doi:10.1371/journal.pgen.1007230)

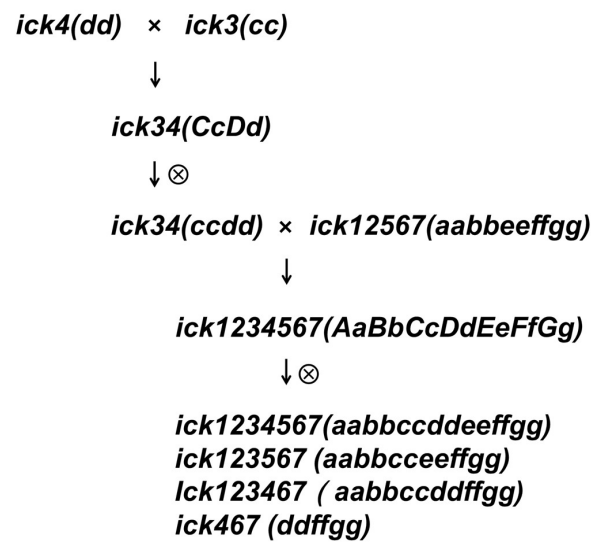

**Figure S2.** Crosses used to produce various *ick* mutants from *ICK* single mutants and *ick12567* mutant.

Supplement: S2 Fig — (PDF) [file pgen.1007230.s002.pdf]
