# Supplementary material for: Arabidopsis ICK/KRP cyclin-dependent kinase inhibitors function to ensure the formation of one megaspore mother cell and one functional megaspore per ovule
Source: PLoS Genet. 2018 Mar 7;14(3):e1007230. doi: 10.1371/journal.pgen.1007230 (PMC5858843; doi:10.1371/journal.pgen.1007230)
Supplement: S3 Fig — (A) Gene-specific primers for ICK1, ICK2, ICK3, ICK4, ICK5, ICK6 and ICK7 were used to confirm the genotype of ICK septuple mutant with WT genomic DNA as a control. (B) Analysis of ICK transcripts in the WT (first lane) and septuple mutant (second lane), with WT genomic DNA as a control (third lane). Gene-specific primers were used for amplifying the full-length sequences of ICK1 to ICK7. Actin was used as a control (the last row). (PDF) [file pgen.1007230.s003.pdf]

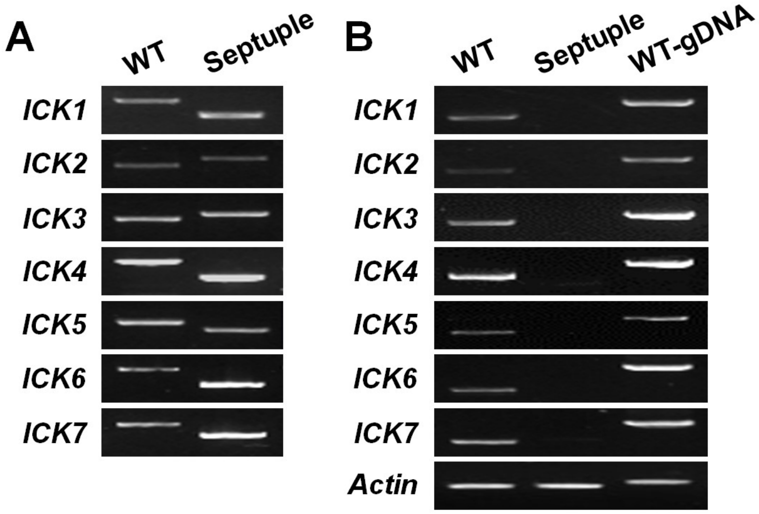

Figure S3. Genotyping and RT-PCR of *ick* septuple mutant.

(**A**) Gene-specific primers for *ICK1*, *ICK2*, *ICK3*, *ICK4*, *ICK5*, *ICK6* and *ICK7* were used to confirm the genotype of *ICK* septuple mutant with WT genomic DNA as a control.

(**B**) Analysis of *ICK* transcripts in the WT (first lane) and septuple mutant (second lane), with WT genomic DNA as a control (third lane). Gene-specific primers were used for amplifying the full-length sequences of *ICK1* to *ICK7*. *Actin* was used as a control (the last row).
