# Supplementary material for: Arabidopsis ICK/KRP cyclin-dependent kinase inhibitors function to ensure the formation of one megaspore mother cell and one functional megaspore per ovule
Source: PLoS Genet. 2018 Mar 7;14(3):e1007230. doi: 10.1371/journal.pgen.1007230 (PMC5858843; doi:10.1371/journal.pgen.1007230)
Supplement: S4 Fig — (A) Seedlings of WT, ick12567 and septuple mutants at 26 days after planting in soil. (B) Fresh seedling weight of 21-day-old WT, ick12567 and septuple mutants. For each line, 3 pots each with 5 plants were used in the analysis. The averages and standard deviations are shown. (C—E) Leaf area (C), blade length (D) and leaf length/width ratio (E) of 21-day-old WT, ick12567 and septuple mutant plants (6 plants from each line were used). The 1st, 2nd, 3rd and 4th pairs of true leaves of WT, ick12567 and septuple mutant were separated, placed on a flat surface, and their photos were taken with a digital camera. The leaf area, leaf blade length and width were measured using ImageJ software. The leaf length/width ratio for each leaf was obtained from its length and width. The averages and standard errors are shown. Data in (B—E) were analyzed using one-way ANOVA and post-hoc Tukey test, and significant differences are indicated by different letters (lower case) at p<0.05 level. (PDF) [file pgen.1007230.s004.pdf]

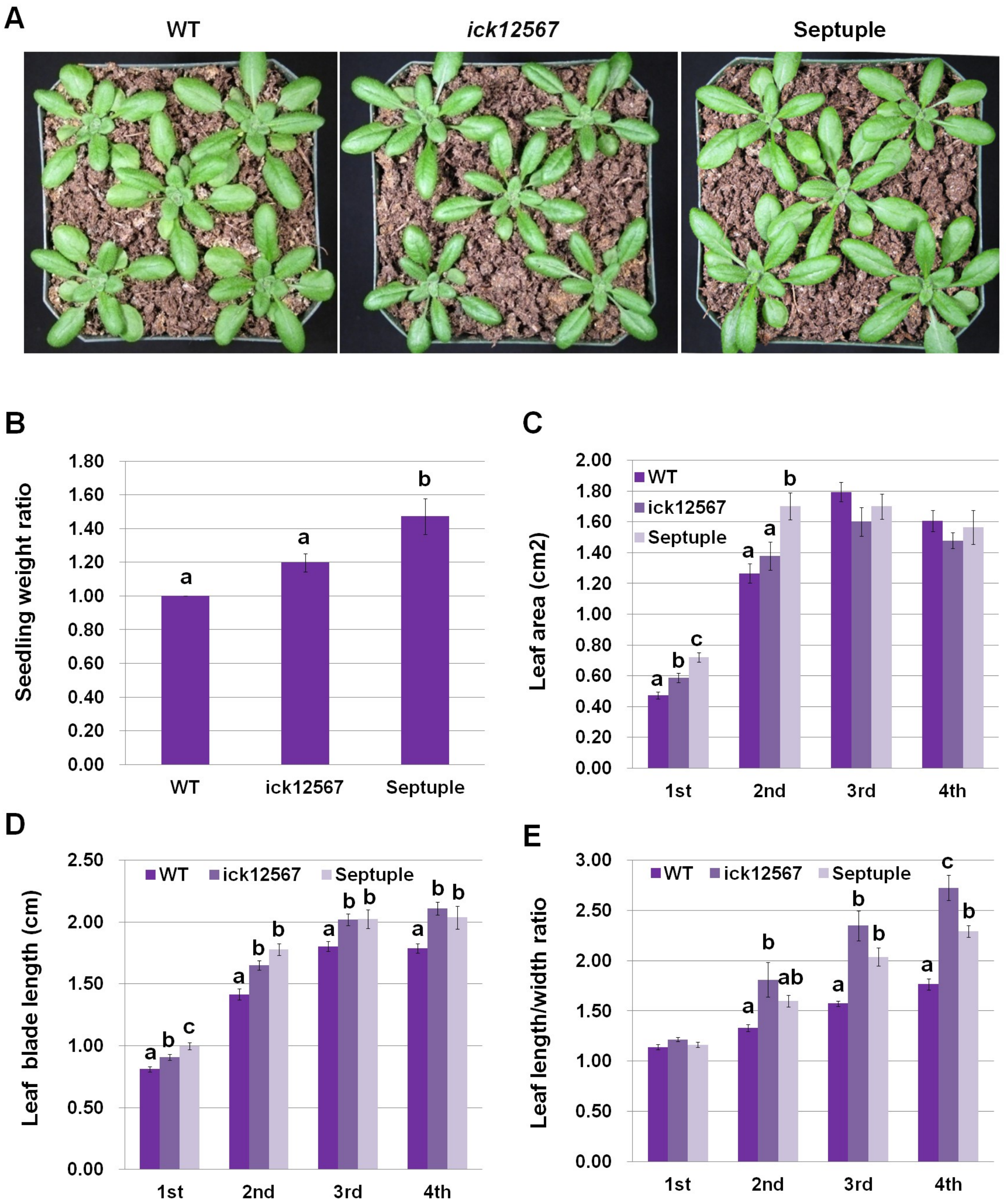

Figure S4. Phenotyping of WT, *ick12567* and septuple mutant plants.

(**A**) Seedlings of WT, *ick12567* and septuple mutants at 26 days after planting in soil.

(**B**) Fresh seedling weight of 21-day-old WT, *ick12567* and septuple mutants. For each line, 3 pots each with 5 plants were used in the analysis. The averages and standard deviations are shown.

(**C - E**) Leaf area (**C**), blade length (**D**) and leaf length/width ratio (**E**) of 21-day-old WT, *ick12567* and septuple mutant plants (6 plants from each line were used). The 1st, 2nd, 3rd and 4th pairs of true leaves of WT, *ick12567* and septuple mutant were separated, placed on a flat surface, and their photos were taken with a digital camera. The leaf area, leaf blade length and width were measured using ImageJ software. The leaf length/width ratio for each leaf was obtained from its length and width. The averages and standard errors are shown.

Data in (**B - E**) were analyzed using one-way ANOVA and post-hoc Tukey test, and significant differences are indicated by different letters (lower case) at $p<0.05$ level.
