# Supplementary material for: Arabidopsis ICK/KRP cyclin-dependent kinase inhibitors function to ensure the formation of one megaspore mother cell and one functional megaspore per ovule
Source: PLoS Genet. 2018 Mar 7;14(3):e1007230. doi: 10.1371/journal.pgen.1007230 (PMC5858843; doi:10.1371/journal.pgen.1007230)
Supplement: S5 Fig — Reciprocal crosses (indicated as female X male) were made between the WT and septuple mutant. The length of fully-elongated siliques was measured. In each treatment, four plants with 4 siliques for each plant were used. The averages and standard deviations are showed. Data were analyzed using one-way ANOVA and post-hoc Tukey test, and significant differences are indicated by different letters (upper case) at p<0.01 level. (PDF) [file pgen.1007230.s005.pdf]

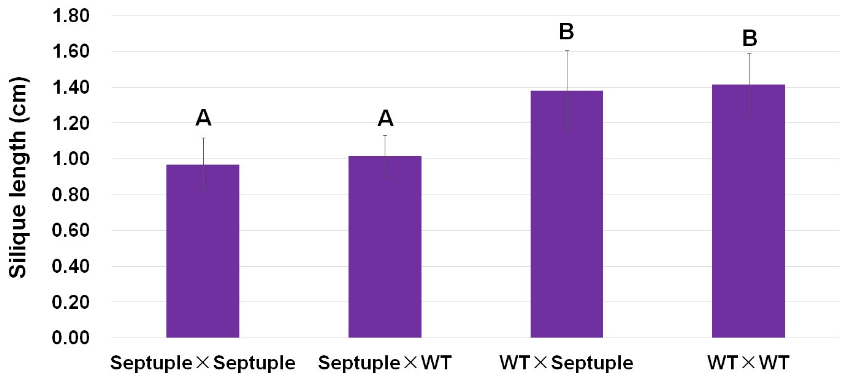

Figure S5. Reciprocal crosses to determine the transmission of the short silique phenotype through the parents. Reciprocal crosses (indicated as female X male) were made between the WT and septuple mutant. The length of fully-elongated siliques was measured. In each treatment, four plants with 4 siliques for each plant were used. The averages and standard deviations are showed. Data were analyzed using one-way ANOVA and post-hoc Tukey test, and significant differences are indicated by different letters (upper case) at $p<0.01$ level.
