# Supplementary material for: Arabidopsis ICK/KRP cyclin-dependent kinase inhibitors function to ensure the formation of one megaspore mother cell and one functional megaspore per ovule
Source: PLoS Genet. 2018 Mar 7;14(3):e1007230. doi: 10.1371/journal.pgen.1007230 (PMC5858843; doi:10.1371/journal.pgen.1007230)
Supplement: S6 Fig — An ICK4 genomic fragment (including 2040 bp before ATG, 1028 bp of the coding region and 717 bp of the region after the STOP codon) was introduced into the septuple mutant. Multiple independent lines were identified that showed complementation of the silique length phenotypes. Two independent T2 lines are presented here. PCR was used to determine the genotypes of the WT, septuple mutant and complementation lines. For each gene, duplex PCR was performed to detect the WT allele (with gene-specific primers for the full-length coding region) and the T-DNA allele using (with a gene-specific primer and a left border primer of T-DNA). The complementation lines are the same as the septuple mutant except for the presence of the WT ICK4 band. (PDF) [file pgen.1007230.s006.pdf]

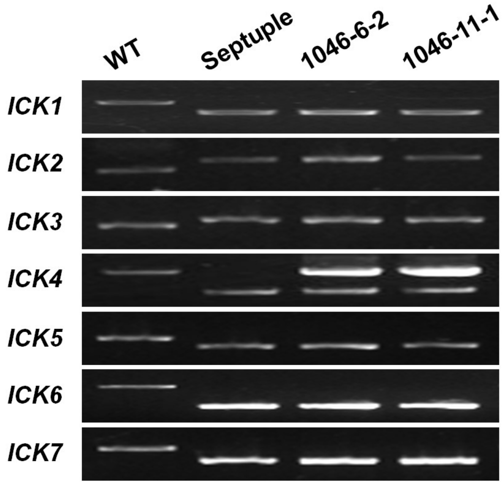

Figure S6. Genotyping of WT, septuple mutant and complementation lines with a genomic *ICK4* fragment. An *ICK4* genomic fragment (including 2040 bp before ATG, 1028 bp of the coding region and 717 bp of the region after the STOP codon) was introduced into the septuple mutant. Multiple independent lines were identified that showed complementation of the silique length phenotypes. Two independent T2 lines are presented here. PCR was used to determine the genotypes of the WT, septuple mutant and complementation lines. For each gene, duplex PCR was performed to detect the WT allele (with gene-specific primers for the full-length coding region) and the T-DNA allele using (with a gene-specific primer and a left border primer of T-DNA). The complementation lines are the same as the septuple mutant except for the presence of the WT *ICK4* band.
