# Supplementary material for: Arabidopsis ICK/KRP cyclin-dependent kinase inhibitors function to ensure the formation of one megaspore mother cell and one functional megaspore per ovule
Source: PLoS Genet. 2018 Mar 7;14(3):e1007230. doi: 10.1371/journal.pgen.1007230 (PMC5858843; doi:10.1371/journal.pgen.1007230)
Supplement: S7 Fig — (A) ICK4 transcript analysis. cDNAs synthesized from total RNAs were used to detect the ICK4 transcripts in WT, septuple mutant and two complementation lines (T2 generation). Actin (ACT8) was amplified as a control. (B) Silique length of WT, septuple mutant and the two complementation lines. Four plants and in each plant the 6th -15th siliques from the bottom of the main florescence were measured. The averages and standard deviations are showed. (C) Opened siliques of WT, septuple mutant and two complementation lines showing the seed development. Scale bar = 1 mm. (D) Number of seeds per silique (the 6th -15th siliques per line in the main inflorescence from each plant). The averages and standard deviations are showed. (E) Number of aborted ovules per silique. Fully extended siliques (10 siliques for each line) were opened and aborted ovules counted under a dissecting microscope. The averages and standard deviations are shown. Data in (B, D, E) were analyzed using one-way ANOVA and post-hoc Tukey test, and significant differences are indicated by different letters (upper case) at p<0.01 level. (PDF) [file pgen.1007230.s007.pdf]

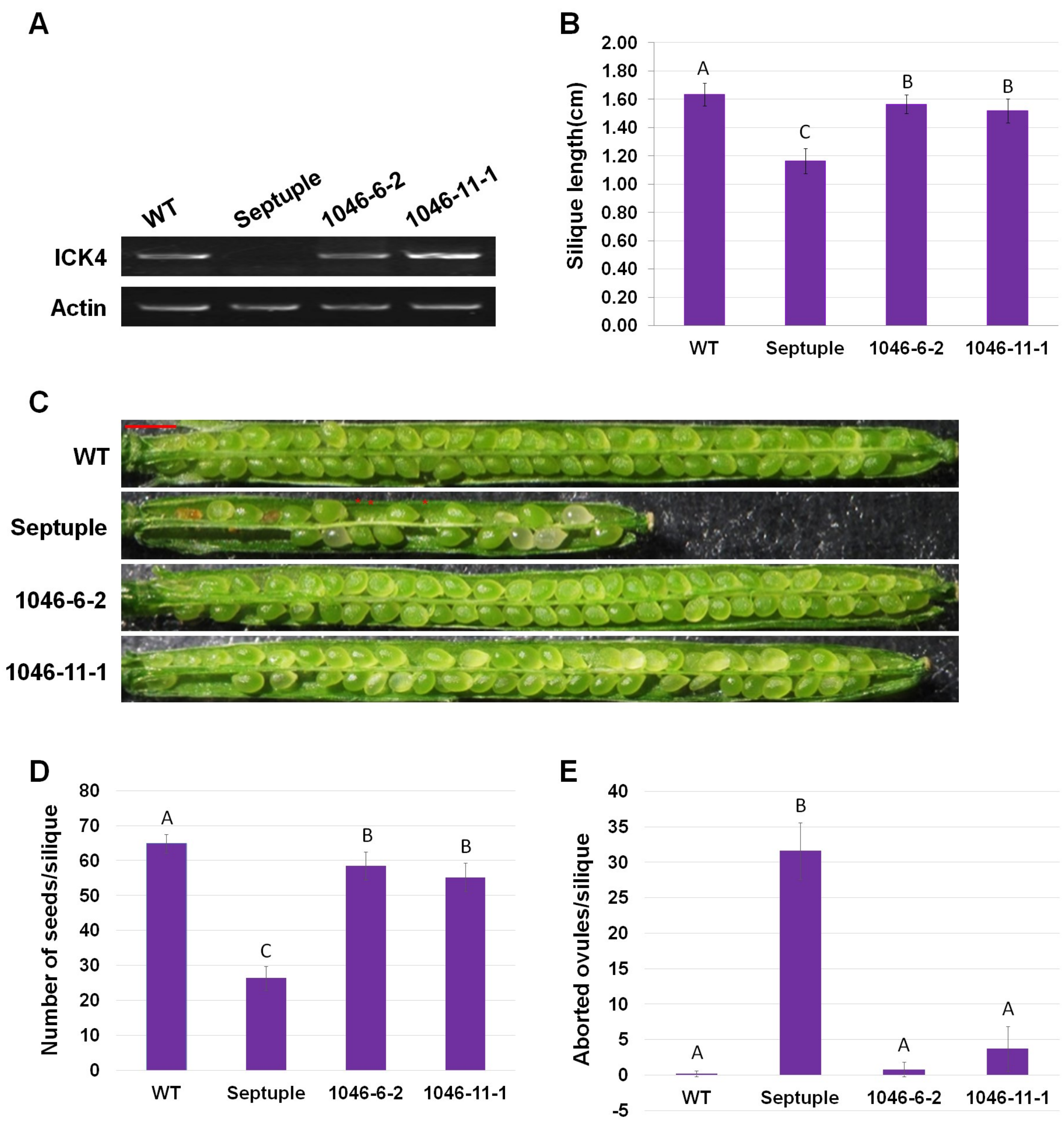

Figure S7. RT-PCR and phenotyping of WT, septuple mutant and complementation lines with a genomic *ICK4* fragment.

(**A**) *ICK4* transcript analysis. cDNAs synthesized from total RNAs were used to detect the *ICK4* transcripts in WT, septuple mutant and two complementation lines (T2 generation). *Actin (ACT8)* was amplified was a control.

(**B**) Silique length of WT, septuple mutant and the two complementation lines. Four plants and in each plant the 6th -15th siliques from the bottom of the main florescence were measured. The averages and standard deviations are showed.

(**C**) Opened siliques of WT, septuple mutant and two complementation lines showing the seed development. Scale bar = 1 mm.

(**D**) Number of seeds per silique (the 6th -15th siliques per line in the main inflorescence from each plant). The averages and standard deviations are showed.

(**E**) Number of aborted ovules per silique. Fully extended siliques (10 siliques for each line) were opened and aborted ovules counted under a dissecting microscope. The averages and standard deviations are shown.

Data in (**B, D, E**) were analyzed using one-way ANOVA and post-hoc Tukey test, and significant differences are indicated by different letters (upper case) at $p<0.01$ level.
