# Supplementary material for: Arabidopsis ICK/KRP cyclin-dependent kinase inhibitors function to ensure the formation of one megaspore mother cell and one functional megaspore per ovule
Source: PLoS Genet. 2018 Mar 7;14(3):e1007230. doi: 10.1371/journal.pgen.1007230 (PMC5858843; doi:10.1371/journal.pgen.1007230)
Supplement: S8 Fig — An ICK7 genomic fragment was introduced into the septuple mutant. Many transformants (6/40) with normal silique length were observed and the analysis of two independent transformants (1243–4 and 1243–23) is shown here. PCR was used to determine the genotypes of the WT, septuple mutant and transformants with ICK7 genomic fragment. For each gene, duplex PCR was performed to detect the WT allele (with gene-specific primers for the full-length coding region) and the T-DNA allele using (a gene-specific primer and a left border primer of T-DNA). The transformants are the same as the septuple mutant except for the presence of the WT ICK7 band. (PDF) [file pgen.1007230.s008.pdf]

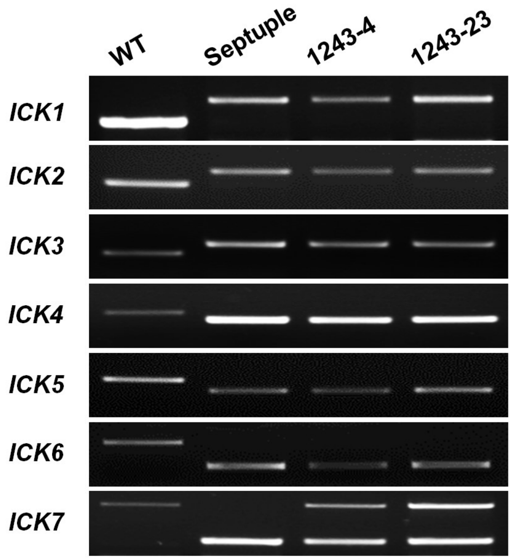

Figure S8. Genotyping of WT, septuple mutant and complementation lines with a genomic *ICK7* fragment. An *ICK7* genomic fragment was introduced into the septuple mutant. Many transformants (6/40) with normal silique length were observed and the analysis of two independent transformants (1243-4 and 1243-23) is shown here. PCR was used to determine the genotypes of the WT, septuple mutant and transformants with *ICK7* genomic fragment. For each gene, duplex PCR was performed to detect the WT allele (with gene-specific primers for the full-length coding region) and the T-DNA allele using (a gene-specific primer and a left border primer of T-DNA). The transformants are the same as the septuple mutant except for the presence of the WT *ICK7* band.
