# Supplementary material for: Arabidopsis ICK/KRP cyclin-dependent kinase inhibitors function to ensure the formation of one megaspore mother cell and one functional megaspore per ovule
Source: PLoS Genet. 2018 Mar 7;14(3):e1007230. doi: 10.1371/journal.pgen.1007230 (PMC5858843; doi:10.1371/journal.pgen.1007230)
Supplement: S9 Fig — (A) ICK7 transcript analysis. cDNAs synthesized from total RNAs were used to detect the ICK7 transcripts in WT, septuple mutant and two transformants with ICK7 genomic fragment. Actin (ACT8) was amplified as a control. (B) Silique length of WT, septuple mutant and the two transformants. Ten siliques (6th-15th) from the bottom of the main florescence were measured for each plant. The averages and standard deviations are showed. (C) Opened siliques of WT, septuple mutant and the two transformants showing the seed development. Scale bar = 1 mm. (D) Number of seeds per silique (8 siliques (7th-14th) in the main inflorescence from each plant). The averages and standard deviations are showed. (E) Number of aborted ovules per silique. Fully extended siliques (8 siliques for each plant) were opened and aborted ovules counted under a dissecting microscope. The averages and standard deviations are shown. Data in (B, D, E) were analyzed using one-way ANOVA and post-hoc Tukey test, and significant differences are indicated by different letters (upper case) at p<0.01 level. (PDF) [file pgen.1007230.s009.pdf]

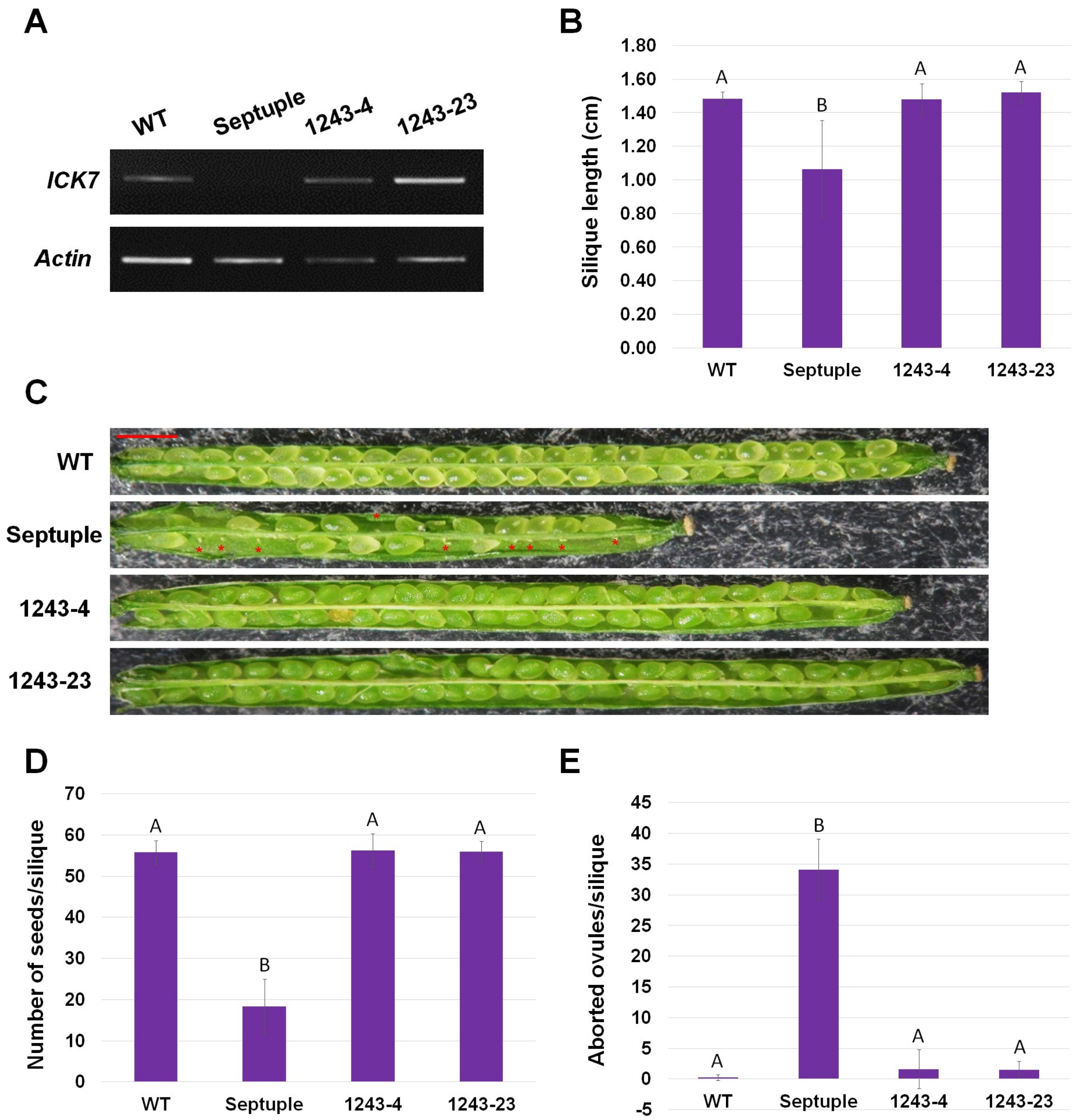

Figure S9. RT-PCR and phenotyping of WT, septuple mutant and transformants with *ICK7*.

(**A**) *ICK7* transcript analysis. cDNAs synthesized from total RNAs were used to detect the *ICK7* transcripts in WT, septuple mutant and two transformants with *ICK7* genomic fragment. *Actin (ACT8)* was amplified was a control.

(**B**) Silique length of WT, septuple mutant and the two transformants. Ten siliques (6th-15th) from the bottom of the main florescence were measured for each plant. The averages and standard deviations are showed.

(**C**) Opened siliques of WT, septuple mutant and the two transformants showing the seed development. Scale bar = 1 mm.

(**D**) Number of seeds per silique (8 siliques (7th-14th) in the main inflorescence from each plant). The averages and standard deviations are showed.

(**E**) Number of aborted ovules per silique. Fully extended siliques (8 siliques for each plant) were opened and aborted ovules counted under a dissecting microscope. The averages and standard deviations are shown.

Data in (**B, D, E**) were analyzed using one-way ANOVA and post-hoc Tukey test, and significant differences are indicated by different letters (upper case) at $p<0.01$ level.
