# Supplementary material for: Arabidopsis ICK/KRP cyclin-dependent kinase inhibitors function to ensure the formation of one megaspore mother cell and one functional megaspore per ovule
Source: PLoS Genet. 2018 Mar 7;14(3):e1007230. doi: 10.1371/journal.pgen.1007230 (PMC5858843; doi:10.1371/journal.pgen.1007230)
Supplement: S10 Fig — Gene-specific primers for ICK1, ICK2, ICK3, ICK4, ICK5, ICK6 and ICK7 genes, and a T-DNA left border primer were used to confirm the genotypes of WT, ick4, ick467, ick123467 and septuple mutants. (PDF) [file pgen.1007230.s010.pdf]

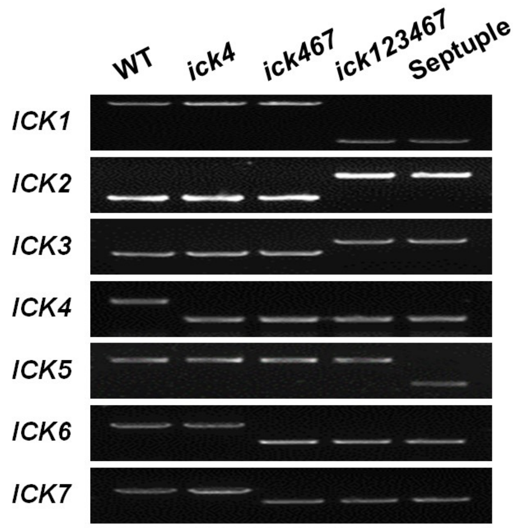

Figure S10. Genotyping of WT, *ick4*, *ick467*, *ick123467* and septuple mutants.
Gene-specific primers for *ICK1*, *ICK2*, *ICK3*, *ICK4*, *ICK5*, *ICK6* and *ICK7* genes, and a T-DNA left border primer were used to confirm the genotypes of WT, *ick4*, *ick467*, *ick123467* and septuple mutants.
