# Supplementary material for: Arabidopsis ICK/KRP cyclin-dependent kinase inhibitors function to ensure the formation of one megaspore mother cell and one functional megaspore per ovule
Source: PLoS Genet. 2018 Mar 7;14(3):e1007230. doi: 10.1371/journal.pgen.1007230 (PMC5858843; doi:10.1371/journal.pgen.1007230)
Supplement: S11 Fig — (A) Analysis of ICK transcripts in the WT (the first lane), ick4 (the second lane), ick467 (the third lane), ick123467 (the fourth lane) and septuple mutant (the fifth lane), with WT genomic DNA as a control (the last lane). Gene-specific primers were used for amplifying the full-length sequences of ICK1 to ICK7. Actin was used as a control (last row). (B) Silique length. The average and standard deviation are shown for the length of fully extended siliques (4 plants per line with 8 siliques from each plant measured). (C) Opened siliques showing silique length and aborted ovules of WT, ick4, ick467, ick123467 and septuple mutants. (D) Number of seeds per silique (4 plants per line with 6 siliques from each plant). The averages and standard deviations are shown. (E) Number of aborted ovules per silique. Fully extended siliques were opened and aborted ovules counted under a dissecting microscope (4 plants per line with 6 siliques from each plant). The averages and standard deviations are shown. Data in (B, D, E) were analyzed using one-way ANOVA and post-hoc Tukey test, and significant differences are indicated by different letters (upper case) at p<0.01 level. (PDF) [file pgen.1007230.s011.pdf]

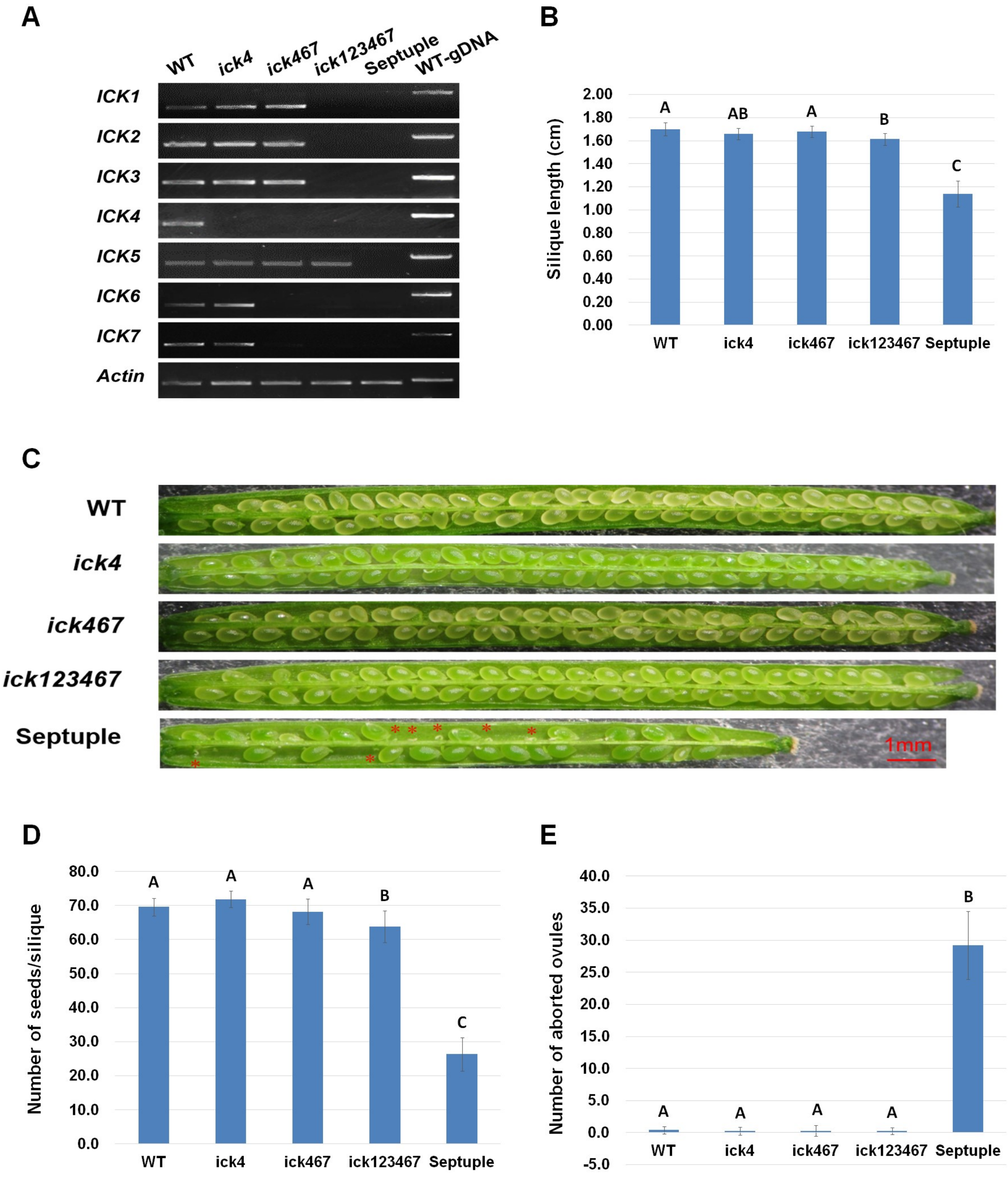

Figure S11. RT-PCR and phenotyping of WT, *ick4*, *ick467*, *ick123467* and septuple mutants.

(**A**) Analysis of *ICK* transcripts in the WT (the first lane), *ick4* (the second lane), *ick467* (the third lane), *ick123467* (the fourth lane) and septuple mutant (the fifth lane), with WT genomic DNA as a control (the last lane). Gene-specific primers were used for amplifying the full-length sequences of *ICK1* to *ICK7*. *Actin* was used as a control (last row).

(**B**) Silique length. The average and standard deviation are shown for the length of fully extended siliques (4 plants per line with 8 siliques from each plant measured).

(**C**) Opened siliques showing silique length and aborted ovules of WT, *ick4*, *ick467*, *ick123467* and septuple mutants.

(**D**) Number of seeds per silique (4 plants per line with 6 siliques from each plant). The averages and standard deviations are shown.

(**E**) Number of aborted ovules per silique. Fully extended siliques were opened and aborted ovules counted under a dissecting microscope (4 plants per line with 6 siliques from each plant). The averages and standard deviations are shown.
Data in (**B, D, E**) were analyzed using one-way ANOVA and post-hoc Tukey test, and significant differences are indicated by different letters (upper case) at $p<0.01$ level.
