# Supplementary material for: Arabidopsis ICK/KRP cyclin-dependent kinase inhibitors function to ensure the formation of one megaspore mother cell and one functional megaspore per ovule
Source: PLoS Genet. 2018 Mar 7;14(3):e1007230. doi: 10.1371/journal.pgen.1007230 (PMC5858843; doi:10.1371/journal.pgen.1007230)
Supplement: S12 Fig — (A) WT and septuple ovules at stage 2-II were surveyed under a DIC microscope and determined for the number of enlarged MMC-like cells prior to meiosis. Number of ovules counted: WT = 129 and mutant = 149. (B) Number of MMCs undergoing meiosis as revealed by immunostaining with an antibody against DMC1, a protein specifically expressed during meiosis, but not at MMC and FG1 stages (for images see Fig 4). Number of ovules counted: WT = 59 and mutant = 60. (C) Number of MMCs estimated based on the callose deposition following meiosis. The callose deposition could be attributed to one, two, three or more MMCs since a typical pattern is produced as the result of meiosis from one MMC. Number of ovules counted: WT = 127 and mutant = 227. (PDF) [file pgen.1007230.s012.pdf]

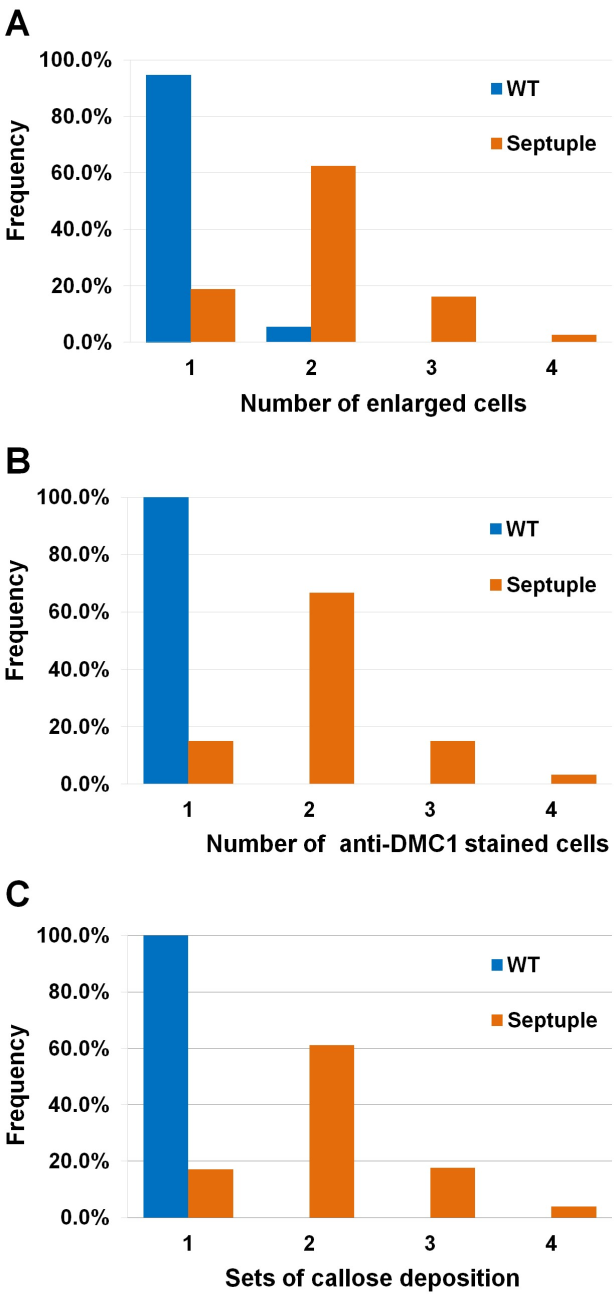

Figure S12. Number of megaspore mother cells determined by DIC, DMC1 immunostaining and callose deposition patterns.

(**A**) WT and septuple ovules at stage 2-II were surveyed under a DIC microscope and determined for the number of enlarged MMC-like cells prior to meiosis. Number of ovules counted: WT = 129 and mutant = 149.

(**B**) Number of MMCs undergoing meiosis as revealed by immunostaining with an antibody against DMC1, a protein specifically expressed during meiosis, but not at MMC and FG1 stages (for images see Figure 4). Number of ovules counted: WT = 59 and mutant = 60.

(**C**) Number of MMCs estimated based on the callose deposition following meiosis. The callose deposition could be attributed to one, two, three or more MMCs since a typical pattern is produced as the result of meiosis from one MMC. Number of ovules counted: WT = 127 and mutant = 227.
