# Supplementary material for: Arabidopsis ICK/KRP cyclin-dependent kinase inhibitors function to ensure the formation of one megaspore mother cell and one functional megaspore per ovule
Source: PLoS Genet. 2018 Mar 7;14(3):e1007230. doi: 10.1371/journal.pgen.1007230 (PMC5858843; doi:10.1371/journal.pgen.1007230)
Supplement: S13 Fig — Developing ovules were prepared and immunostained with an antibody against ASY1, which is specifically expressed during meiosis. (A—C) WT ovules at MMC (A), meiosis (B) and FG1 (C) stages. (D—I) Mutant ovules at MMC (D), meiosis (E—H) and FG1 (I) stages. One to four cells in the mutant ovules could be stained with DMC1 (E—H). Scale bars equal 5 μm. (PDF) [file pgen.1007230.s013.pdf]

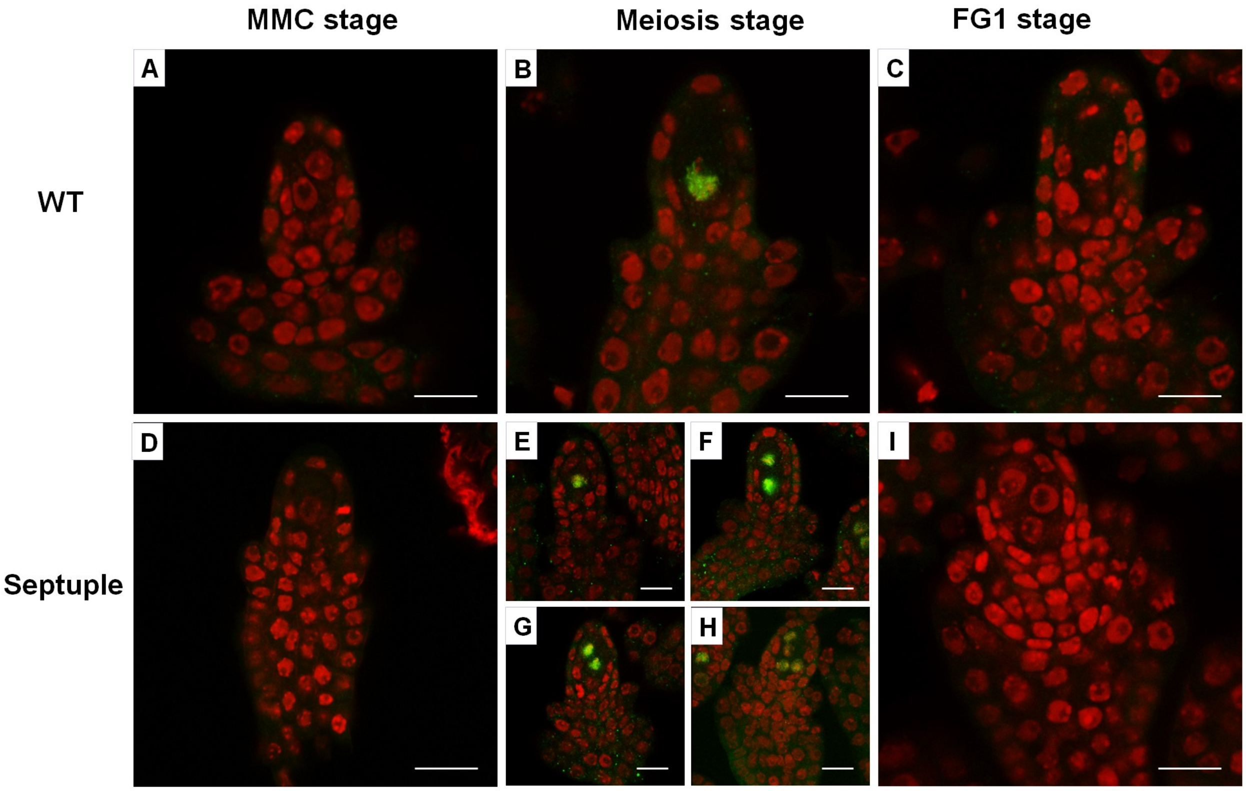

Figure S13. Identification of megaspore mother cells (MMCs) in meiosis by ASY1 immunostaining.

Developing ovules were prepared and immunostained with an antibody against ASY1, which is specifically expressed during meiosis.

(**A - C**) WT ovules at MMC (**A**), meiosis (**B**) and FG1 (**C**) stages.

(**D - I**) Mutant ovules at MMC (**D**), meiosis (**E - H**) and FG1 (**I**) stages. One to four cells in the mutant ovules could be stained with DMC1 (**E - H**).

Scale bars equal 5 μm.
