# Supplementary material for: Arabidopsis ICK/KRP cyclin-dependent kinase inhibitors function to ensure the formation of one megaspore mother cell and one functional megaspore per ovule
Source: PLoS Genet. 2018 Mar 7;14(3):e1007230. doi: 10.1371/journal.pgen.1007230 (PMC5858843; doi:10.1371/journal.pgen.1007230)
Supplement: S14 Fig — Ovules with a single MMC were identified based on callose staining with aniline blue and DIC microscopy. For each ovule, the callose fluorescence image is shown at the top and the DIC image at the bottom. The numbers in the images indicate the 1st to 4th megaspore position from the chalazal to micropylar end. (A—B) WT ovule with one megaspore at the most chalazal position surviving. (C—L) show mutant ovules. (C—D) Mutant ovule with one megaspore at the 4th position surviving. (E—F) Two megaspores at the 1st and 2nd positions surviving. (G—H) Two megaspores at the 1st and 4th positions surviving. (I—J) Two megaspores at the 3rd and 4th positions surviving. (K—L) All four megaspores seemly surviving. Scale bar in (A) is for all images and equals 10 μm. (PDF) [file pgen.1007230.s014.pdf]

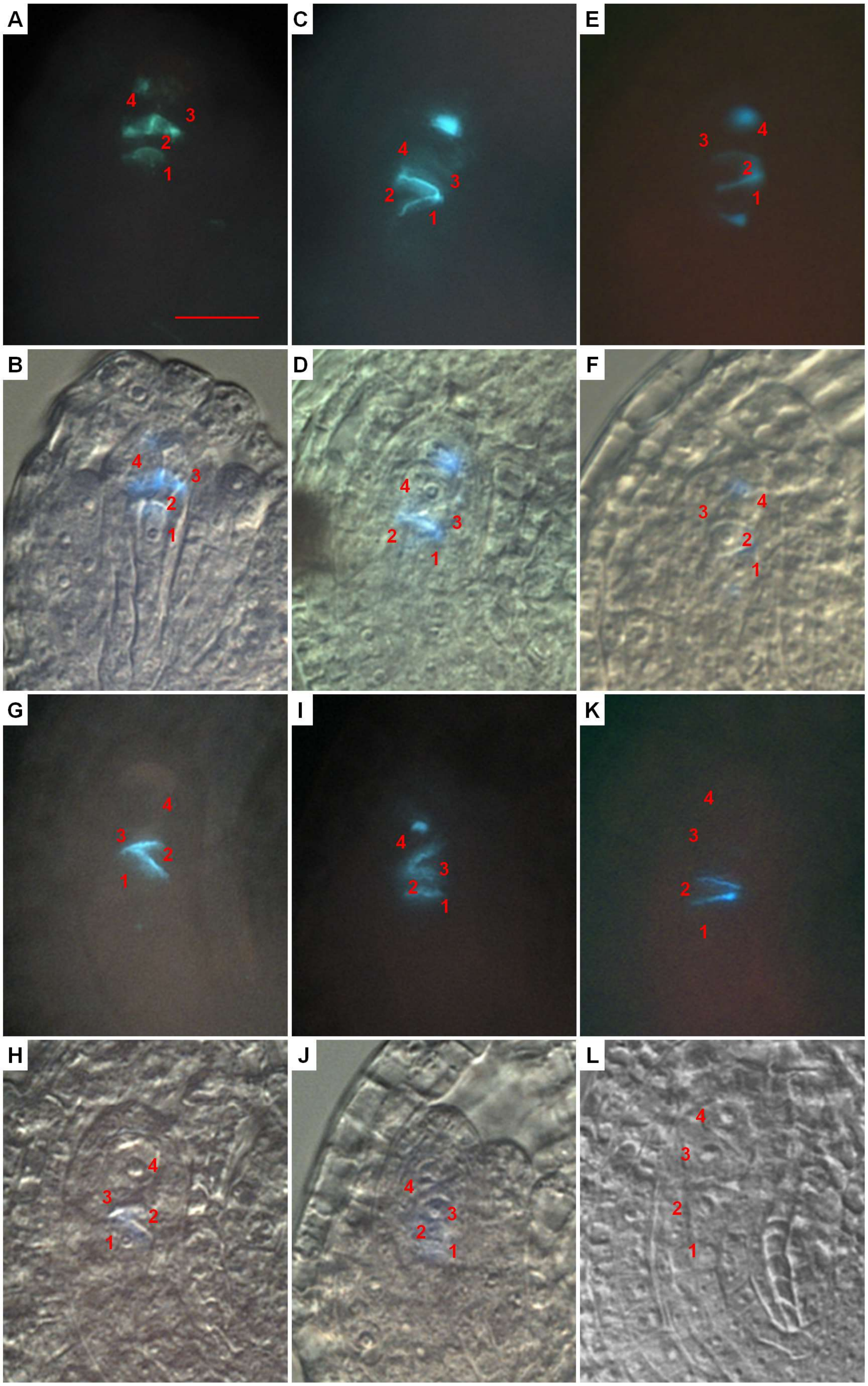

Figure S14. Analysis of surviving megaspores in WT and septuple mutant ovules with one MMC.

Ovules with a single MMC were identified based on callose staining with aniline blue and DIC microscopy. For each ovule, the callose fluorescence image is shown at the top and the DIC image at the bottom. The numbers in the images indicate the 1st to 4th megaspore position from the chalazal to micropylar end.

(**A - B**) WT ovule with one megaspore at the most chalazal position surviving. (**C - L**) show mutant ovules. (**C - D**) Mutant ovule with one megaspore at the 4th position surviving.

(**E - F**) Two megaspores at the 1st and 2nd positions surviving. (G **- H**) Two megaspores at the 1st and 4th positions surviving. (**I - J**) Two megaspores at the 3rd and 4th positions surviving. (**K - L**) All four megaspores seemly surviving. Scale bar in (**A**) is for all images and equals 10 μm.
