# Supplementary material for: Arabidopsis ICK/KRP cyclin-dependent kinase inhibitors function to ensure the formation of one megaspore mother cell and one functional megaspore per ovule
Source: PLoS Genet. 2018 Mar 7;14(3):e1007230. doi: 10.1371/journal.pgen.1007230 (PMC5858843; doi:10.1371/journal.pgen.1007230)
Supplement: S15 Fig — WT (A) and septuple (B—D) ovules at FG2 stage. The WT ovule had one pair of nuclei. The mutant ovules had two (B), three (C) and four (D) pairs of nuclei, with each pair likely derived from a functional megaspore. The two nuclei in each pair are close to each other and have similar appearance. The boundary between different pairs is often visible (B, D). Different numbers in (B—D) indicate different pairs of nuclei. Scale bar in (A) is for all images equals 10 μm. (PDF) [file pgen.1007230.s015.pdf]

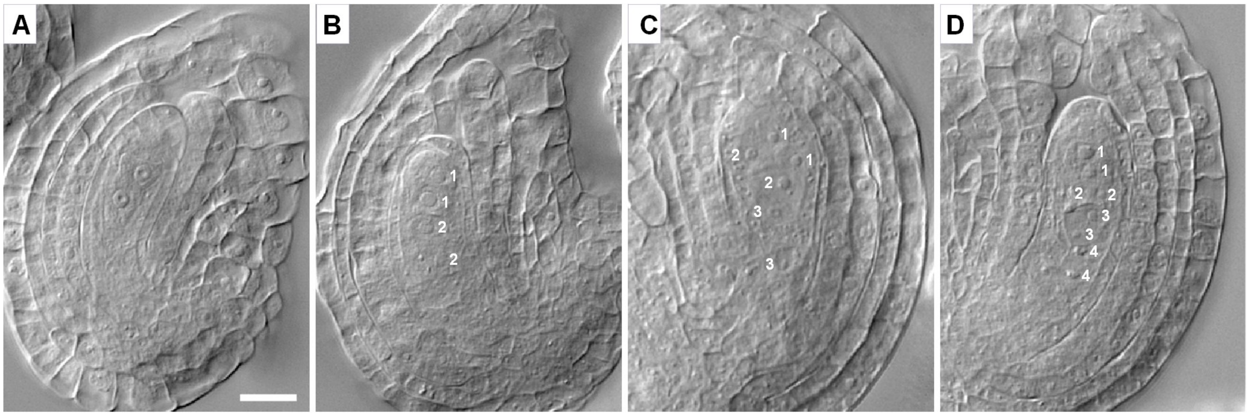

Figure S15. Early megagametogenesis in WT and *ick* septuple mutant.
WT (**A**) and septuple (**B - D**) ovules at FG2 stage. The WT ovule had one pair of nuclei. The mutant ovules had two (**B**), three (**C**) and four (**D**) pairs of nuclei, with each pair likely derived from a functional megaspore. The two nuclei in each pair are close to each other and have similar appearance. The boundary between different pairs is often visible (**B, D**). Different numbers in (**B - D**) indicate different pairs of nuclei. Scale bar in (**A**) is for all images equals 10 μm.
