# Supplementary material for: Arabidopsis ICK/KRP cyclin-dependent kinase inhibitors function to ensure the formation of one megaspore mother cell and one functional megaspore per ovule
Source: PLoS Genet. 2018 Mar 7;14(3):e1007230. doi: 10.1371/journal.pgen.1007230 (PMC5858843; doi:10.1371/journal.pgen.1007230)
Supplement: S1 Table — Mature pollen grains from ten nascent flowers each line were collected and stained with DAPI. Vegetative and sperm cell nuclei were observed and counted under a fluorescence microscope (Leica DM2500) with a 63× (NA = 1.40) oil lens. (PDF) [file pgen.1007230.s016.pdf]

Table S1. Analysis of mature pollen grains in WT and *ick* septuple mutant

Line	Pollen grains surveyed	Vegetative nuclei		Generative nuclei		
		0	1	0	1	2
WT	1098	47 (4.4%)	1051 (95.6%)	1 (0.1%)	13 (1.2%)	1084 (98.7%)
Septuple	1145	33 (2.9%)	1112 (97.1%)	63 (5.5%)	52 (4.5%)	1030 (90.0%)

Mature pollen grains from ten nascent flowers each line were collected and stained with DAPI. Vegetative and sperm cell nuclei were observed and counted under a fluorescence microscope (Leica DM2500) with a 63× (NA = 1.40) oil lens.
