# Supplementary material for: Arabidopsis ICK/KRP cyclin-dependent kinase inhibitors function to ensure the formation of one megaspore mother cell and one functional megaspore per ovule
Source: PLoS Genet. 2018 Mar 7;14(3):e1007230. doi: 10.1371/journal.pgen.1007230 (PMC5858843; doi:10.1371/journal.pgen.1007230)
Supplement: S2 Table — The flower buds just before opening were emasculated and left for 1 day. Ovules (from about 15 gynoecia for each line) were prepared and observed under a microscope with DIC optics. If one central cell (secondary) nucleus and one egg nucleus, usually close to each other, could be identified, they were considered as one set. The synergid nuclei were not always recognizable when there were three or four sets of gametes. For the mutant, a large portion (about 47% by a separate analysis) of embryo sac did not contain any observable secondary and egg nuclei, and were not included in this survey. (PDF) [file pgen.1007230.s017.pdf]

Table S2. Observation of female gametes in mature embryo sacs in the WT and *ick* septuple mutant

Line	Embryo sacs analyzed	Sets of central cell and egg nuclei observed			
		1	2	3	4
WT	98	98 (100%)	0	0	0
Septuple	204	111 (54.4%)	78 (38.2%)	11 (5.4%)	4 (2.0%)

The flower buds just before opening were emasculated and left for 1 day. Ovules (from about 15 gynoecia for each line) were prepared and observed under a microscope with DIC optics. If one central cell (secondary) nucleus and one egg nucleus, usually close to each other, could be identified, they were considered as one set. The synergid nuclei were not always recognizable when there were three or four sets of gametes. For the mutant, a large portion (about 47% by a separate analysis) of embryo sac did not contain any observable secondary and egg nuclei, and were not included in this survey.
