# Supplementary material for: Arabidopsis ICK/KRP cyclin-dependent kinase inhibitors function to ensure the formation of one megaspore mother cell and one functional megaspore per ovule
Source: PLoS Genet. 2018 Mar 7;14(3):e1007230. doi: 10.1371/journal.pgen.1007230 (PMC5858843; doi:10.1371/journal.pgen.1007230)
Supplement: S3 Table — The ovules were checked and those at functional megaspore stage were included in the analysis. They were observed under a microscope with DIC optics. The presence and number of functional megaspores in ovules (from about 15 gynoecia) were counted. (PDF) [file pgen.1007230.s018.pdf]

Table S3. Development of functional megaspore (FM) in ovules of the WT and *ick* septuple mutant.

	**Total ovules**	**Stages of ovule**					
		No nucleus observed	Number of functional megaspores				FG2
			1	2	3	4	
**WT**	62	3 (4.8%)	57 (92.0%)	0	0	0	2 (3.2%)
**Septuple**	106	31 (29.3%)	32 (30.2%)	30 (28.3%)	10 (9.4%)	3 (2.8%)	0

The ovules were checked and those at functional megaspore stage were included in the analysis. They were observed under a microscope with DIC optics. The presence and number of functional megaspores in ovules (from about 15 gynoecia) were counted.
