# Supplementary material for: Arabidopsis ICK/KRP cyclin-dependent kinase inhibitors function to ensure the formation of one megaspore mother cell and one functional megaspore per ovule
Source: PLoS Genet. 2018 Mar 7;14(3):e1007230. doi: 10.1371/journal.pgen.1007230 (PMC5858843; doi:10.1371/journal.pgen.1007230)
Supplement: S4 Table — Three days after flowers opened, ovules were collected and prepared for DIC observation. Only the ovules containing at least one developing embryo were included (about 20 gynoecia from each line used). All the WT ovules had one endosperm compartment with uniform endosperm nuclei, while some mutant ovules had two endosperm compartments. Nuclei resembling the secondary nucleus were also present in the endosperm of the mutant. The secondary nucleus might have divided into several nuclei, which were recognizable against the background of endosperm nuclei (See Fig 8C). The nuclei that were close to each other physically and looked similar morphologically were considered to be derived from one original secondary nucleus. “+1CC”, “+2CC” and “+3CC” indicate the number of central cells that are inferred to based on the extra secondary nuclei present. (PDF) [file pgen.1007230.s019.pdf]

Table S4. Analysis of endosperm development and presence of extra central cell nuclei after fertilization in the WT and *ick* septuple mutant

	Total	One endosperm compartment	+1CC	+2CC	+3CC	Two endosperm compartments	+1CC	+2CC
WT	168	168 （100%）	0	0	0	0	0	0
Septuple	347	206 （59.4%）	42 （12.1%）	10 （2.9%）	0 （0）	78 （22.5%）	10 （2.9%）	1 （0.3%）

Three days after flowers opened, ovules were collected and prepared for DIC observation. Only the ovules containing at least one developing embryo were included (about 20 gynoecia from each line used). All the WT ovules had one endosperm compartment with uniform endosperm nuclei, while some mutant ovules had two endosperm compartments. Nuclei resembling the secondary nucleus were also present in the endosperm of the mutant. The secondary nucleus might have divided into several nuclei, which were recognizable against the background of endosperm nuclei (See Figure 8c). The nuclei that were close to each other physically and looked similar morphologically were considered to be derived from one original secondary nucleus. “+1CC”, “+2CC” and “+3CC” indicate the number of central cells that are inferred to based on the extra secondary nuclei present.
