# Supplementary material for: Arabidopsis ICK/KRP cyclin-dependent kinase inhibitors function to ensure the formation of one megaspore mother cell and one functional megaspore per ovule
Source: PLoS Genet. 2018 Mar 7;14(3):e1007230. doi: 10.1371/journal.pgen.1007230 (PMC5858843; doi:10.1371/journal.pgen.1007230)
Supplement: S5 Table — WT and septuple mutant seeds were plated on ½ MS plates. Four days after plating, seedlings were screened under a dissecting microscope for the occurrence of twin seedlings. (PDF) [file pgen.1007230.s020.pdf]

Table S5. Frequency of twin seedlings in the WT and *ick* septuple mutant

	No. of seeds screened	No. of twin seedlings (%)
WT	1379	0 (0%)
Septuple	2760	58 (2.1%)

WT and septuple mutant seeds were plated on ½ MS plates. Four days after plating, seedlings were screened under a dissecting microscope for the occurrence of twin seedlings.
